# Knock-down of LRP/LR influences signalling pathways in late-stage colorectal carcinoma cells

**DOI:** 10.1186/s12885-021-08081-3

**Published:** 2021-04-09

**Authors:** Leila Vania, Gavin Morris, Eloise Ferreira, Stefan F. T. Weiss

**Affiliations:** School of Molecular and Cell Biology, University of the Witwatersrand, Private Bag 3, Wits 2050, Johannesburg, Republic of South Africa

**Keywords:** Colorectal cancer, Small interfering RNAs, Apoptosis, 37 kDa/67 kDa laminin receptor, LRP/LR, Telomerase, Proteomics, Therapeutics

## Abstract

**Background:**

The 37 kDa/67 kDa laminin receptor (LRP/LR) is involved in several tumourigenic-promoting processes including cellular viability maintenance and apoptotic evasion. Thus, the aim of this study was to assess the molecular mechanism of LRP/LR on apoptotic pathways in late stage (DLD-1) colorectal cancer cells upon siRNA-mediated down-regulation of LRP/LR.

**Methods:**

siRNAs were used to down-regulate the expression of LRP/LR in DLD-1 cells which was assessed using western blotting and qPCR. To evaluate the mechanistic role of LRP/LR, proteomic analysis of pathways involved in proliferation and apoptosis were investigated. The data from the study was analysed using a one-way ANOVA, followed by a two-tailed student’s *t*-test with a confidence interval of 95%.

**Results:**

Here we show that knock-down of LRP/LR led to significant changes in the proteome of DLD-1 cells, exposing new roles of the protein. Moreover, analysis showed that LRP/LR may alter components of the MAPK, p53-apoptotic and autophagic signalling pathways to aid colorectal cancer cells in continuous growth and survival. Knock-down of LRP/LR also resulted in significant decreases in telomerase activity and telomerase-related proteins in the DLD-1 cells.

**Conclusions:**

These findings show that LRP/LR is critically implicated in apoptosis and cell viability maintenance and suggest that siRNA-mediated knock-down of LRP/LR may be a possible therapeutic strategy for the treatment of colorectal cancer.

**Supplementary Information:**

The online version contains supplementary material available at 10.1186/s12885-021-08081-3.

## Background

Colorectal cancer has been ranked the 3rd most common cancer type in 2018, and was found to be among the top five most frequent locations for cancer development in men and women globally [1]. Due to the increasing prevalence and mortality rates of colorectal cancer, it is crucial to develop a novel treatment strategy to combat this disease.

Recently, research into the 37 kDa/ 67 kDa laminin receptor (LRP/LR) has gained a large amount of interest as it plays several physiological roles within cells [2]. These include ribosomal processing and protein translation [3], nuclear structure maintenance [4, 5], and linking of ribosomes to microtubules [6]. In addition to these physiological roles, LRP/LR is also found to have pathological roles in several diseases [7–10].

The receptor has been shown to play a major role in cancer since it is often over-expressed on the surface of several cancer cells [11–13]. This over-expression is said to enhance the adhesion of tumour cells to the basement membrane and disseminate these tumours – both fundamental events of metastasis [11, 14] as well as promote tumour angiogenesis [15]. Moreover, LRP/LR is also involved in critical maintenance of tumourigenic cell viability. Several studies found that the down-regulation of LRP/LR through siRNA technology results in the induction of apoptosis in various cancer cell lines [16, 17], including early (SW-480) and late (DLD-1) colorectal cancer cells [18].

In addition to LRP/LR’s roles in cancer, the receptor has been found to contribute to telomerase activity in breast cancer cells [19]. Telomerase is principally responsible for maintaining and elongating telomeres [20] however, it is also important in cellular senescence and immortalization thus, playing a significant role in ageing and tumourigenesis [21]. Studies have shown that for telomerase activity to be regulated in cells, telomerase reverse transcriptase (hTERT) must be activated [22]. hTERT is the catalytic subunit of telomerase responsible for the addition of TTAGGG telomeric repeats to the ends of chromosomes while also acting as a transcriptional activator for several proliferative genes [23]. In addition to its role as the core regulator of telomerase activity, and consequently, proliferative potential, studies have found that there is a direct association between hTERT and an increased resistance to apoptosis [24]. In addition to telomerase and hTERT, telomeres are also linked to telomere-related proteins which work with telomeres to protect and “cap” genomic DNA by preventing damage and degradation [25]. Two important telomere-related proteins are the telomeric repeat-binding factor-1 (TRF-1) and − 2 (TRF-2) which telomere sequences are found to bind directly [25]. Once the telomere repeats bind to TRF-1 and TRF-2, they are responsible for directly protecting telomeres as well as regulating telomere length [25].

Interestingly, it has been shown that there is a relationship between LRP/LR and telomerase [10]. More importantly, the two proteins have interlinked roles in various diseases such as cancer and neurodegenerative disorders [26]. Moreover, additional studies have revealed that the knock-down of LRP/LR significantly reduces telomerase activity [19] and this may therefore, may be a potential therapeutic intervention for cancer.

Although these findings have given some insight into LRP/LR and its mechanism in cancer and apoptosis, more information is still needed to fully understand the role that LRP/LR plays in the evasion of apoptosis in cancer. Thus, these outcomes encouraged the question of whether siRNA-mediated down-regulation of LRP/LR influences the expression of specific proteins involved in apoptosis and other cell signalling pathways in colorectal carcinoma cells.

## Methods

### Cell cultivation

The DLD-1 colorectal cancer cell line was obtained from Fox Chase Cancer Centre (FCCC)/ Institute for Cancer Research in 2015. The cell line was authenticated upon arrival by IDEXX Bioresearch through species-specific PCR and marker STR profile contamination tests. In addition, the DLD-1 cells were periodically stained with DAPI to test for any mycoplasma contamination [refer to Vania et al. (2018) for original confocal microscopy images of cells]. Please see supplementary section for detailed cell cultivation procedure.

### Transfection procedure

All transfections were performed as per Vania at el (2018). Please refer to Table S1-S3 for full protocols as well as target sequences of Human-RPSA and control siRNA-RLUC used for down-regulation of LRP/LR.

### SDS-PAGE and Western blotting

To determine the levels of several proteins upon siRNA-mediated down-regulation of LRP/LR, western blotting was performed. The Trans-Blot® Turbo™ Transfer System was used for electroblotting with set parameters at 25 V for 30 min. Following this, the membranes were blocked for an hour using 3% (w/v) BSA in 1 X PBS-Tween (0.1% (v/v) Tween 20 and PBS). Thereafter, the membranes were incubated overnight in the appropriate primary antibody diluted in blocking buffer [IgG1-iS18 (1:1000), anti-β actin peroxidase, (1:1000), rabbit anti-TRF-1 (1:1000), rabbit anti-TRF-2 (1:1000), rabbit anti-pTERT (1:1000) and mouse anti-hTERT (1:1000)]. The membranes then underwent three 10-min washes in 1 X PBS-Tween. Thereafter, the membranes were incubated for 1 h at room temperature in the appropriate secondary antibody with a horseradish peroxidase (HRP) conjugate which was diluted in 3% (w/v) BSA in 1 X PBS-Tween. This was followed by three more 10-min washes in 1 X PBS-Tween before the chemiluminescent substrate was added and proteins were detected using the ChemiDoc™ imaging machine. The 42 kDa β-actin antibody was used for the detection of β-actin which served as a loading control. The Bio-Rad Image Lab 5.1 Image acquisition and analysis software was used to analyse the blot features and capture the image data.

### Cell cycle analysis – Flow cytometry

Please see supplementary data section for detailed method.

### Telomerase activity assay - quantitative polymerase chain reaction (qPCR)

#### Sample preparation

A total of 750,000 DLD-1 cells were seeded per well in 6-well tissue culture plates prior to transfection for 72 h. The cells were then harvested and centrifuged at 1500 x g for 10 min. The resultant pellets were resuspended in 250 μl of CHAPS lysis buffer followed by 30-min incubation on ice. Thereafter, samples were centrifuged for 20 min at 15000 x g at 4 °C in a microcentrifuge where the resulting supernatant was snap frozen on dry ice. The protein concentration was then quantified using Nanodrop spectroscopy (Nanodrop® ND-1000) and the proteins were diluted to 500 ng/μl.

#### Telomerase activity detection

Relative telomerase activity was quantified by the TRAPeze® RT1 Telomerase detection Kit (Merck Millipore), following the manufacturers protocol. All samples were then subjected to experimental analysis by qPCR accompanied by positive control HEK293 cell extracts with confirmed telomerase activity. Three negative controls were used: a minus telomerase control, consisting of only CHAPS Lysis Buffer; a no template control consisting of only nuclease free water, and lastly, a heat-treated telomerase negative control. Thereafter, 10 μl of each 500 ng/μl sample was incubated at 85 °C for 10 min prior to detection for telomerase inactivation. All reactions were performed in triplicate in 96-well qPCR plates. The samples were placed in the CFX Maestro™ thermocycler instrument and qPCR was performed with the following cycling parameters: One cycle of 37 °C for 30 min, 95 °C for 2 min and 45 cycles of 95 °C for 15 s, 59 °C for 60 s and 45 °C for 10 s. Telomerase activity was thereafter calculated from the standard curve generated by 1:10 serial dilutions (20–0.0002 amoles) of the provided TSR8 control template as per Merck Millipore instructions. Negative controls were included. The data was then analysed with CFX Maestro™ software version 1.0.

### Proteome profiler antibody arrays™

To determine whether proteins involved in the apoptotic and MEK/ERK signalling pathways are affected post siRNA-mediated down-regulation of LRP, Proteome Profiler Antibody arrays were performed as per the supplier’s instructions (R&D systems). The intensity score of each duplicated array spot was measured with the ImageLab (version 5.1) software and the average intensity was calculated by subtracting the averaged background signal and PBS spots (negative control). The identity and the respective coordinates of all the antibodies on the arrays can be found in the supplementary data section.

### Quantification of mRNA expression levels – quantitative reverse transcriptase polymerase chain reaction (RT-qPCR)

#### RNA extraction

A total of 750,000 DLD-1 cells were seeded per well in 6-well tissue culture plates prior to transfection for 72 h. To determine mRNA expression levels, RNA was first extracted from the DLD-1 samples. The extraction procedure followed the Quick-RNA™ MiniPrep kit (manufacturer’s protocol). The resultant sample concentrations were determined using Nanodrop spectroscopy (Nanodrop® ND-1000).

Thereafter, a 1% (w/v) agarose gel containing a nucleic acid gel stain was used for gel electrophoresis (agarose, in 1 x TBE buffer) to evaluate the RNA integrity. The samples were loaded with 6 x loading dye and a Low range DNA ladder was used as a molecular weight marker. The DNA was separated at 100 V for approximately 30 min in TBE buffer. The RNA was visualized using ChemiDoc™ system in order to view total/intact RNA as well as any DNA contamination (Refer to Supplementary data).

#### cDNA synthesis

Once the absence of DNA contamination was confirmed, the samples were converted into cDNA. The SensiFAST™ cDNA synthesis kit was used as per the manufacturer’s instructions. Thereafter, qPCR was performed using the CFX Maestro™ thermo cycler instrument with the following parameters: One cycle of 25 °C (primer annealing) for 10 min, one cycle of 42 °C (reverse transcription) for 15 min, one cycle of 48 °C (additional reverse transcription for highly structured RNA) for 15 min and one cycle of 85 °C (inactivation) for 5 min. The resultant samples were stored at − 20 °C for mRNA quantification.

### mRNA expression level quantification - quantitative polymerase chain reaction (RT-qPCR)

To quantify mRNA expression levels, qPCR was performed using the SensiFAST SYBR™ No-ROX kit as per the manufacturer’s protocol. The samples were placed in the CFX Maestro™ thermo cycler with the following parameters: One cycle at 95 °C for3 minutes, 45 cycles at 95 °C for 15 s, one cycle T_m_ of gene for 30 s, melt curve: one cycle at 95 °C for 10 s, 65 °C for 1 min and one cycle at 72 °C for 30 s. Refer to the Supplementary data section for the melt peaks of each gene.

Thereafter, the average C_q_ value from technical repeats was used for calculations. To acquire reliable comparisons of gene expression levels between samples reference genes used such as *GAPDH* (Glyceraldehyde 3-phosphate dehydrogenase) and *ACTB* (β-actin). Normalisation with stably expressed reference genes as internal controls, known as the comparative C_q_ or the ΔΔC_q_ method, was used for the normalisation of mRNA data. Reverse transcriptase controls (RTC’s) were also subtracted from all samples for further normalisation. Refer to the supplementary data section for primer sequences, qPCR melt curves and peaks for each gene.

### Sequential window Acquisition of all Theoretical Mass Spectra (SWATH MS)

#### Sample preparation

A total of 750,000 DLD-1 cells were seeded per well in 6-well tissue culture plates prior to transfection for 72 h. The cells were then harvested and centrifuged at 1500 x g for 10 min. Cell pellets were resuspended in 200 μl of lysis buffer [1% SDS, 100 mM Tris-HCl, pH 8.0, MS grade H_2_O] per pellet. Thereafter, cells were sonicated on ice for 9 pulses (10 s per pulse) followed by centrifugation at 15000 x g for 10 min to clear cell debris. Cell lysates were then incubated with 25 units (1 μl of stock – 2500 units in 100 μl) of benzonase per 0.5 million cells and at 2 mM MgCl_2_ at 37 °C for 30 min. This was followed by another centrifugation at 15000 x g for 10 min. The supernatant was collected, and the concentration determined using a BCA assay. Protein solutions were then reduced using 10 mM DTT for 30 min at 37 °C and alkylated using 40 mM IAA for 30 min in the dark.

#### Selection of differentially expressed proteins

Differentially expressed proteins were detected via the Skyline external tool, MS Stats. Thereafter, the MS Stats output list of differentially expressed proteins was further filtered so that only entries fitting the following criteria remained: Minimum fold change ≥2 and Maximum adjusted *p*-value ≤0.01.

Please see supplementary data section for a detailed description of full SWATH-MS method.

### Statistical evaluation

A one-way ANOVA followed by a two-tailed student’s *t*-test with a confidence interval of 95% was used as a means of analysing and confirming the data. To further validate the data, the Bonferroni post-hoc test was applied, with *p*-values of less than 0.05 considered to be significant. The statistical analysis was performed using the Microsoft® Excel 365 statistical programme.

## Results

Successful knock-down of LRP/LR was performed as per Vania et al. (2018) and confirmed before subsequent experiments (see Figs. S1-S3) [18]. Moreover, due to LRP/LR’s role in cell viability maintenance, cell cycle analysis was performed in order to obtain insight into the effect of LRP/LR knock-down as well as to confirm the occurrence of apoptosis. Upon Human-RPSA transfection of DLD-1 cells for 3-days, there was a significant 15% increase in the number of dead cells in the sub G0/G1 stage (apoptotic stage) and 17.4% decrease in G0/G1 phase, indicating the occurrence of apoptosis (Fig. S4). Although this result was significant, it was not a substantial increase. Since apoptosis is a time-dependent process where some alterations may take longer than others to show an effect [27], it was decided to extend the transfection period by 48 h, for a total of 5 days to ensure that the period of apoptotic activity was not missed. Upon transfection of DLD-1 cells with Human-RPSA for 5 days, there was a significant 41% increase in the number of dead cells in the sub G0/G1 stage with a 43% decrease in G0/G1 phase, indicating a higher percentage of cells undergoing apoptosis (Fig. S4b). It is noteworthy that there was no cell cycle arrest in any other phase of the cell cycle. esiRNA-RLUC and PCA acted as the negative and positive controls, respectively (see supplementary data section).

### siRNA-mediated knock-down significantly affects telomere-related proteins in late stage colorectal cancer cells

An increased level of hTERT is found to increase telomerase activity and as a result, enhance tumourigenesis [19]. In addition, TERT phosphorylation is a pre-requisite for telomerase activity to occur and has been found to correlate to increased telomerase activity [28]. Taken together, these factors incited the question of whether siRNA-mediated knock-down of LRP/LR will influence hTERT and phospho-TERT protein expression levels as well as telomerase activity in DLD-1 cells.

**Fig. 1:**
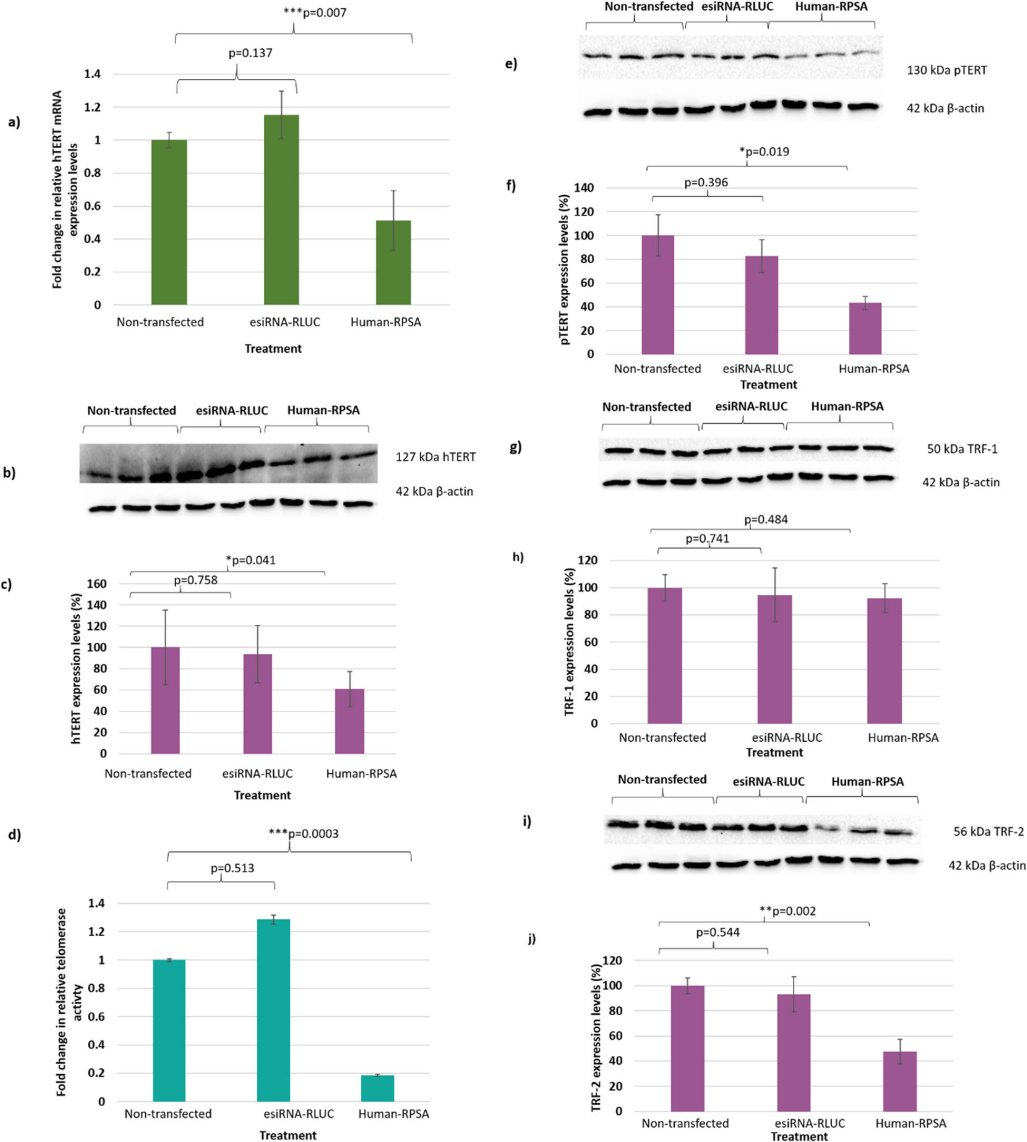
Effect of siRNA-mediated knock-down of LRP/LR on telomerase and telomere-related proteins in late (DLD-1) colorectal cancer cells. (**a**) Upon transfection with Human-RPSA siRNA, relative hTERT mRNA expression levels decreased by 0.5-fold, when compared to non-transfected cells. esiRNA-RLUC served as the negative control. (**b**) Western blot showing a decrease in hTERT protein expression levels upon Human-RPSA siRNA transfection, in comparison to non-transfected cells. (**c**) Densitometric analysis of hTERT protein expression levels show a significant decrease by 0.4-fold in Human-RPSA siRNA transfected cells, when compared to non-transfected cells which were set to 100%. (**d**) Upon Human-RPSA siRNA transfection, telomerase activity significantly decreased by 0.8-fold. esiRNA-RLUC served as the negative control. This data represents three biological replicates with two technical repeats with newly transfected samples each time. (**e**) Western blot showing a decrease in pTERT protein expression levels upon Human-RPSA siRNA transfection, in comparison to non-transfected cells. (**f**) Densitometric analysis of pTERT protein expression levels reveal a significant decrease of 0.6-fold in Human-RPSA siRNA transfected cells, when compared to non-transfected cells which were set to 100%. (**g**) Western blot shows no change in TRF-1 protein expression levels upon Human-RPSA siRNA transfection, when compared to non-transfected cells. (**h**) This was confirmed through densitometric analysis where non-transfected cells were set to 100% and compared to Human-RPSA siRNA transfected cells. (**i**) Western blot showing a decrease in TFR-2 protein expression levels upon Human-RPSA siRNA transfection, in comparison to non-transfected cells. (**j**) Densitometric analysis of TRF-2 protein expression levels show that TRF-2 protein expression levels significantly decrease by 0.5-fold in Human-RPSA siRNA transfected cells, when compared to non-transfected cells which were set to 100%. β-actin was used as the loading control and the graphs are representative of an average of experiments performed in triplicate. *p < 0.05, **p < 0.01, ***p < 0.001, significant: The One-way ANOVA and a Bonferroni corrected post-hoc *t*-test was used for statistical analysis. Full-length blots are presented in Supplementary Figs. S6-S10)

It was found that once the DLD-1 cells were transfected with the Human-RPSA siRNA, hTERT mRNA and protein expression levels were significantly decreased by 0.5-fold (Fig. 1a) and 0.4-fold (Figs. 1b and c), respectively, when compared to non-transfected cells. Moreover, siRNA-mediated knock-down of LRP/LR resulted in a significant decrease of 0.8-fold in telomerase activity, (Fig. 1d) and a significant 0.6-fold decrease in pTERT protein expression levels, when compared to non-transfected cells (Figs. 1e and f).

Telomere sequences are directly bound to the telomeric repeat-binding factor-1 (TRF-1) and − 2 (TRF-2) where both proteins are found to protect and regulate telomere length [29]. Since these proteins play a role in telomerase and cancer, this prompted investigations on how siRNA-mediated knock-down of LRP/LR would affect these telomere-related proteins. When DLD-1 cells were transfected with the Human-RPSA siRNA, it was found that the protein expression levels of TRF-1 was unaffected (Figs. 1g and h), while TRF-2 levels were significantly decreased by 0.5-fold (Figs. 1i and j), in comparison to non-transfected cells.

Previous studies have shown that LRP/LR plays a role in the MAPK/ERK 1/2 cellular survival pathway as well as apoptotic pathways [30] thus, we wanted to confirm these findings. Proteome Profiler Antibody Arrays™ were used to analyse the pathways.

**Fig. 2:**
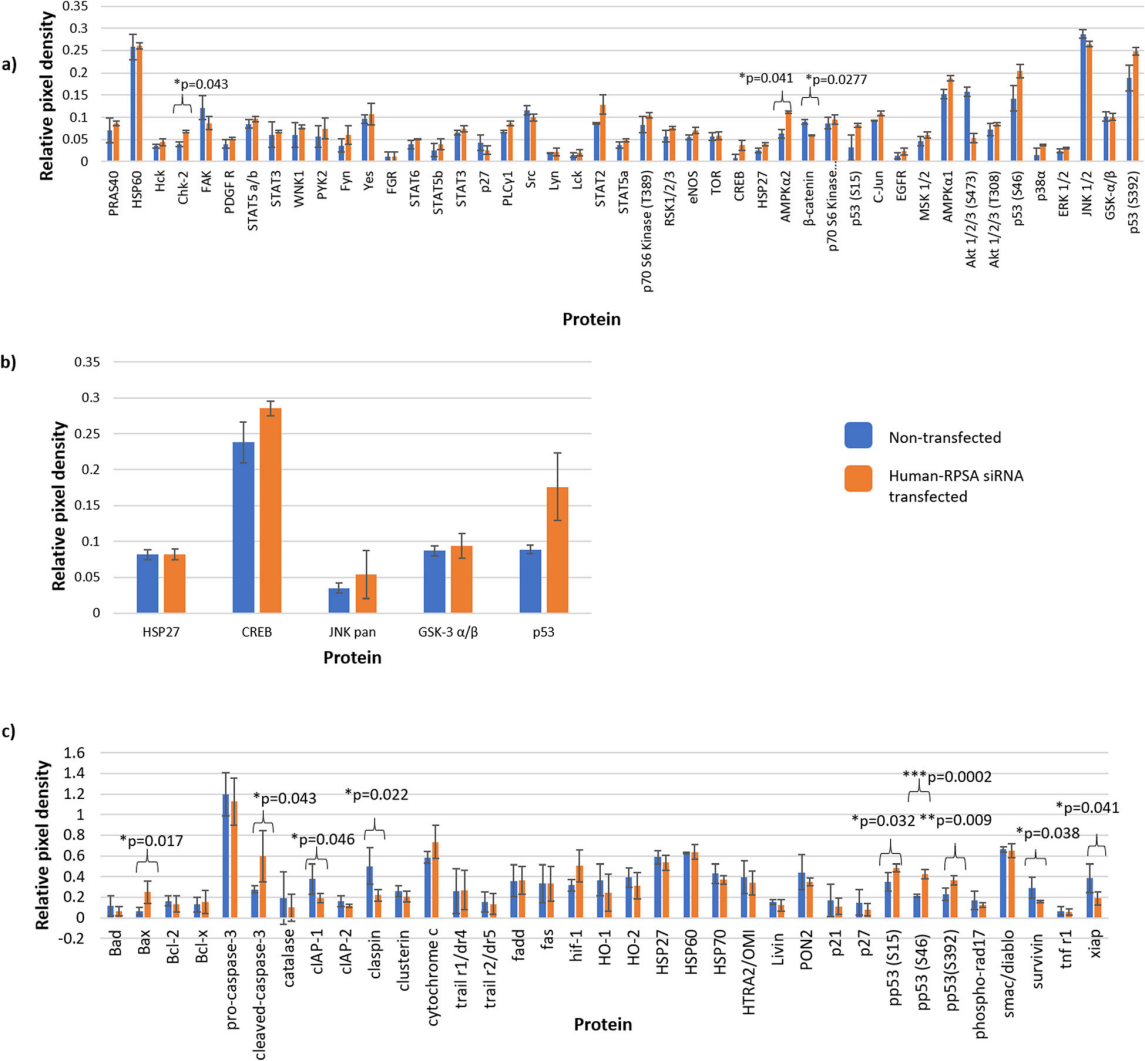
Proteins found to be affected upon siRNA-mediated knock-down of LRP/LR using the Proteome Profiler Antibody Arrays™ in late (DLD-1) stage colorectal cancer cells. (**a**) Densitometric analysis of the phospho-MAPK Proteome Profiler Antibody Array™ revealed that several proteins were affected upon siRNA-mediated knock-down of LRP/LR. When compared to non-transfected cells, Chk-2, β-catenin and AMPKα2 were found to have significant differences. The following proteins namely: CREB, p53, FAK, AMPK α1, Akt 1/2/3, STAT2 and p27 also displayed differences in expression however, these differences were found to be non-significant. (**b**) Densitometric analysis of the MAPK Proteome Profiler Antibody Array™ showed that CREB, GSK-3α/β and p53 were up-regulated once LRP/LR was down-regulated however, this up-regulation was found to be non-significant. **c**) Non-transfected and transfected DLD-1 cell lysates were incubated on the Human Apoptotic Proteome Profiler Antibody membrane arrays. Upon densitometric analysis, it was found that several anti-apoptotic proteins were decreased while several pro-apoptotic proteins were increased, including p53 phosphorylated sites, confirming the result of the MAPK array. All data shown are from 10-min exposure times. Signals for each protein are displayed as a pair of spots, with three pairs of dark reference spots on the lower left, upper right and upper left corners for alignment. PBS spots (designated regions that had consistent background with no capture sites) were used as the negative control where each value was subtracted from PBS and divided by its corresponding reference spot. All graphs represent experiments performed in duplicate with two technical repeats. *p < 0.05, **p < 0.01, ***p < 0.001, significant: The One-way ANOVA was significant, and a Bonferroni corrected post-hoc *t*-test was performed

A Human phospho-MAPK array kit containing 43 kinase antibody capture sites was first used to determine the role of LRP/LR in the MAPK pathway. Upon siRNA-mediated knock-down of LRP/LR, densitometric analysis revealed that 9 proteins of the phospho-MAPK pathway were affected however, only 3 of these proteins, Chk-2, β-catenin and AMPKα2, were found to be significant. The remaining 6 proteins including FAK, CREB, AMPKα1 as well as three phosphorylation sites of p53 (Fig. 2a) revealed changes, however upon statistical analysis, these changes were found to be non-significant. In order to confirm the results from this profiler array, a second MAPK array kit was performed, containing 26 MAPK antibody capture sites. It displayed consistent results with the phospho-MAPK array in that CREB and p53 were once again found to be affected (Fig. 2a and b). However, the change in these proteins was also found to be non-significant.

To confirm that LRP/LR plays a role in cell survival through evasion of apoptosis, Human Apoptosis Proteome Profiler Antibody Arrays™ were performed which contained 35 capture antibody sites. It was found that upon siRNA-mediated knock-down of LRP/LR, 11 proteins were affected. Several pro-apoptotic proteins including Bax, cleaved caspase-3 and p53 phosphorylation sites were significantly up-regulated, while anti-apoptotic proteins such as claspin, cIAP1, XIAP and Survivin were significantly down-regulated (Fig. 2c). Cytochrome C and HIF-1α proteins were also affected by siRNA-mediated knock-down of LRP/LR however these changes were found to be non-significant upon densitometric analysis. The membrane arrays as well as the identity and the respective coordinates of all the antibodies on the arrays can be found in the Supplementary Data section (Figs. S11-S14).

The Proteome Profiler Antibody Arrays™ showed favourable results in illustrating that LRP/LR influences proteins involved in the MAPK pathway as well as the apoptotic pathways. However, similar to western blotting, Proteome Profiler Antibody Arrays™ only provide semi-quantitative data. Thus, to confirm the results obtained from the arrays, specific proteins namely: p53, Bcl-2, Bax, and CREB, were chosen for relative mRNA expression level analysis upon siRNA-mediated knock-down of LRP/LR.

**Fig. 3:**
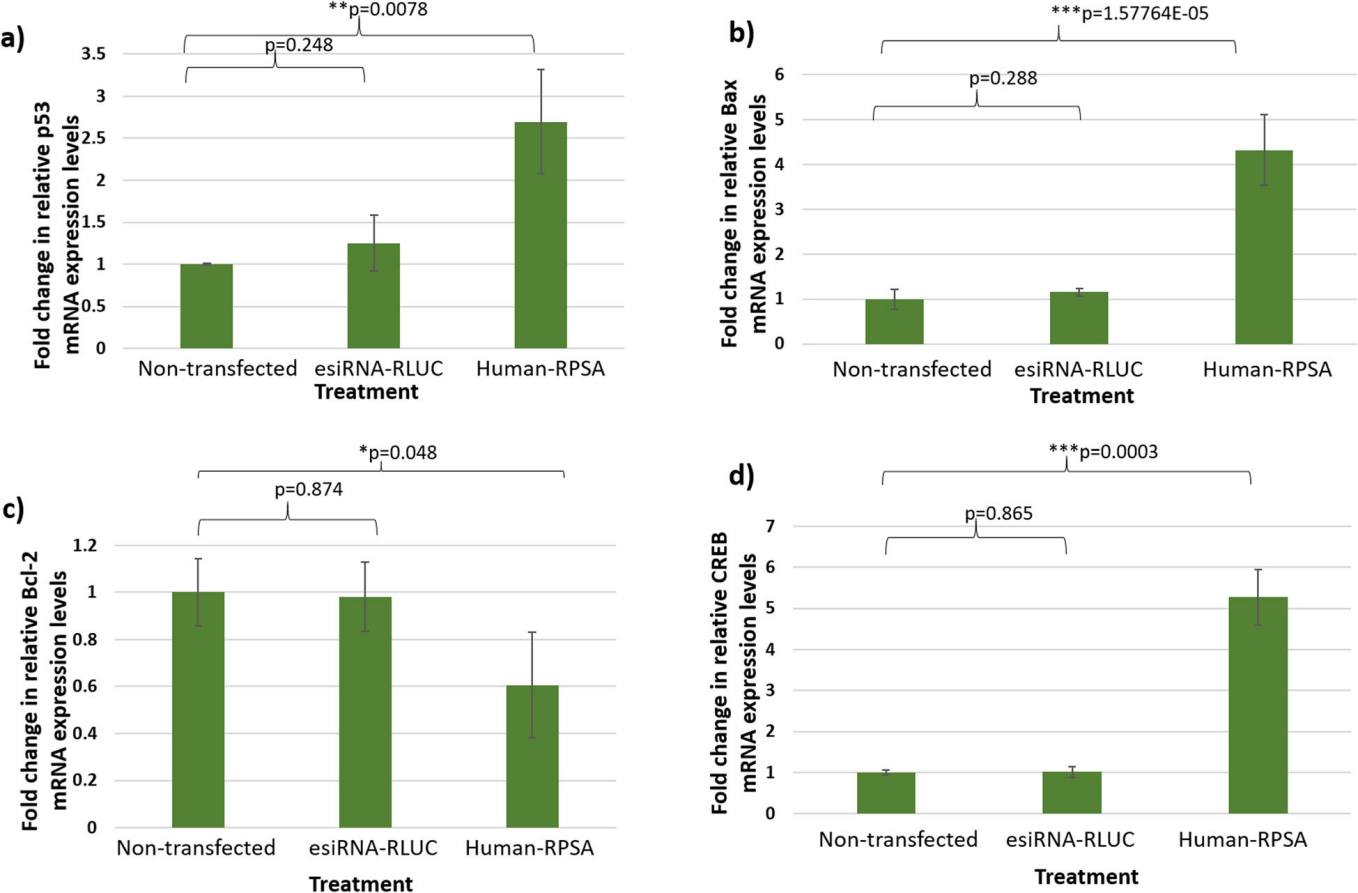
Relative mRNA expression levels of specific proteins in late (DLD-1) colorectal cancer cells. (**a**) Transfection with Human-RPSA siRNA showed that relative p53 mRNA expression levels increased by 1.7-fold, (**b**) Bax increased by 4-fold and (**d**) CREB increased by 4-fold when compared with non-transfected cells. (**c**) Upon transfection with Human-RPSA, relative Bcl-2 mRNA expression levels decreased by 0.4-fold in comparison to non-transfected cells. esiRNA-RLUC served as the negative control for each experiment. All data represent three biological replicates with two technical repeats with newly transfected samples each time. *p < 0.05, **p < 0.01, ***p < 0.001, significant: A One-way ANOVA and a Bonferroni corrected post-hoc *t*-test was performed

It was found that upon siRNA-mediated knock-down of LRP/LR, relative mRNA expression levels of the tumour suppressor gene, p53 and the pro-apoptotic protein, Bax significantly increased by 1.7-fold and 4-fold, respectively, when compared to non-transfected cells (Fig. 3a and b), confirming both the MAPK and Apoptotic Proteome Profiler Antibody Arrays™ (Fig. 2). In addition, siRNA-mediated down-regulation resulted in a decrease in relative mRNA expression levels of the anti-apoptotic protein Bcl-2 in comparison to non-transfected cells (Fig. 3c). Interestingly, the CREB protein was found to be over-expressed in cells transfected with the Human-RPSA siRNA in the Apoptotic Proteome Profiler Array™, thus investigations on whether its mRNA expression level was similarly affected were incited. Upon siRNA-mediated knock-down of LRP/LR, it was found that the relative mRNA expression level of CREB was significantly increased by 4-fold compared to non-transfected cells (Fig. 3d) thus, confirming the results from the array (Figs. 2a and b).

Although a significant change in various proteins was observed in the Proteome Profiler Antibody Arrays™ upon siRNA-mediated knock-down of LRP/LR, the data obtained provided only semi-quantitative results. Sequential Window Acquisition of all Theoretical fragment ion spectra Mass Spectrometry (SWATH-MS) was used to quantify proteins associated with LRP/LR. SWATH-MS is a modified method of data-independent analysis (DDA) MS which integrates quantitative accuracy and consistency together with the ability for deep proteome coverage on a large scale [31]. This therefore allowed for novel screening of proteins affected by siRNA-mediated down-regulation of LRP/LR, enabling insight into the mechanism by which LRP/LR exerts its effects. In order to exclude the effects of the transfection reagent, the negative control esiRNA-RLUC-transfected cells (rather than only the non-transfected) and the Human-RPSA siRNA-transfected samples were compared to one another. Additionally, the non-transfected samples were compared to Human-RPSA siRNA-transfected samples as well as mock-transfected to obtain more insight into the DLD-1 cells (Refer to Fig. S9).

**Fig. 4:**
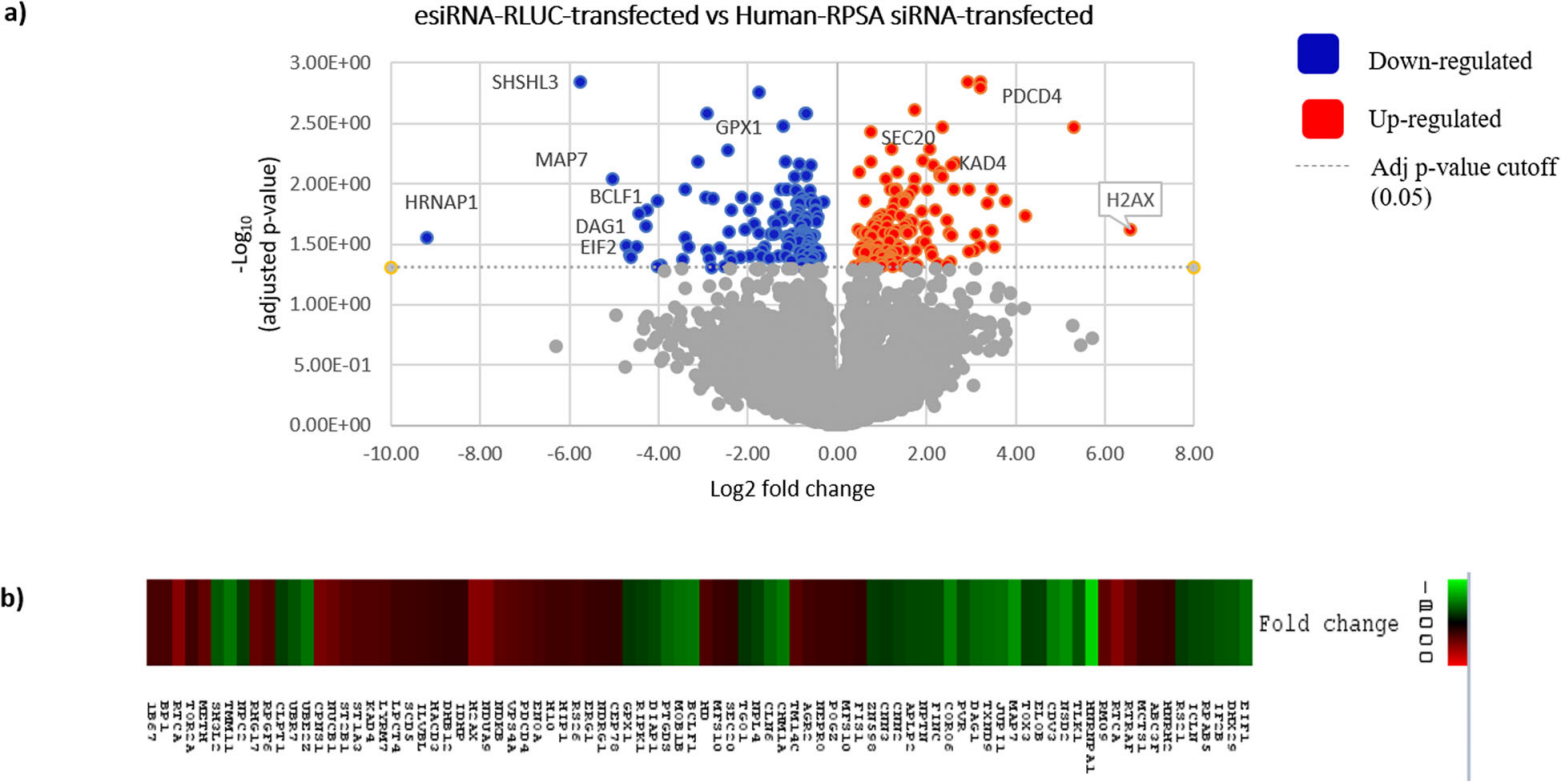
Proteins found to be affected upon siRNA-mediated knock-down of LRP/LR using SWATH-MS based proteomics in late (DLD-1) stage colorectal cancer cells. Only proteins with *p*-values < 0.05 were considered significant. Red dots indicated proteins that were significantly up-regulated, while blue dots show proteins that were significantly down-regulated. (**a**) esiRNA-RLUC-transfected vs Human-RPSA siRNA- samples. It was found that there was a large, significant effect on the proteome of Human-RPSA siRNA transfected samples when they were compared to esiRNA-RLUC negative control samples. (**b**) Heat-map illustrating up- and down-regulated proteins upon siRNA-mediated knock-down of LRP/LR. Proteins which were up-regulated upon transfection with Human-RPSA siRNA are shown in red, while down-regulated proteins are indicated in green

Several proteins were found to be differentially expressed (Fig. 4a and b) and from these proteins, only those that had a 2-fold increase or greater together with a *p*-value < 0.05 were considered significant. As shown in the graphs below, there is a significant change in the proteome of late-stage colorectal cancer cells when transfected with Human-RPSA siRNA. Upon analysis between the Human-RPSA siRNA transfected samples and the esiRNA-RLUC-transfected samples, it was found that 40 proteins were down-regulated while 44 proteins were up-regulated (Fig. 4a). Moreover, a similar result was found when the Human-RPSA siRNA-transfected samples were compared to the non-transfected samples (Fig. S9), confirming the data.

## Discussion

Tumourigenic cells are characteristically found to overexpress the 37 kDa/67 kDa laminin receptor (LRP/LR) compared to their normal cell counterparts [11]. This receptor is involved in promoting several tumourigenic processes such as cell migration and adhesion, cell viability maintenance as well as apoptotic evasion. We have previously shown that the knock-down of LRP/LR via siRNA technology significantly reduced the viability of early (SW-480) and late (DLD-1) stage colorectal cancer cells through the induction of apoptosis [18]. The current study further reveals that the knock-down of LRP/LR leads to the significant decrease in LRP/LR mRNA expression levels (Fig. S3) as well as increases sub G0/G1 apoptotic levels, further confirming successful down-regulation and the occurrence of apoptosis via siRNA technology (Fig. S4).

Thus, to understand the mechanistic role of LRP/LR in proliferation and apoptosis, pathway analysis was performed on the DLD-1 cells after knock-down of LRP/LR. Firstly, LRP/LR’s involvement with telomerase and telomere biology was investigated. When telomeres shorten to a critical length, the dysfunctional telomere is recognised as damaged DNA and senescence is triggered [32]. However, cancerous cells overcome inhibitory pathways induced by DNA damage and senescence by upregulating telomerase activity, leading to the maintenance of unstable telomeres, resulting in cancer formation [32]. Several studies have investigated the expression of hTERT and telomerase activity in non-malignant and malignant colorectal cancer cells – all illustrating that hTERT mRNA expression and telomerase activity are significantly higher in malignant colorectal cancer samples compared to normal colorectal tissue [33, 34].

Upon siRNA-mediated knock-down of LRP/LR, not only was telomerase activity inhibited but hTERT, pTERT and TRF-2 protein expression levels were also decreased (Fig. 1). In addition, a high and positive correlation of R = 0.91 was found between pTERT protein expression levels and telomerase activity post knock-down with the Human-RPSA siRNA. These findings suggest that LRP/LR does indeed influence both hTERT and pTERT levels, and subsequently telomerase activity. p53 has also been found to influence the expression of both TERT and LRP via transcriptional repression in order to limit proliferation [35, 36]. Additionally, p53 affects TERT, telomerase activity as well as TRF-2 for apoptotic induction or cell cycle arrest [37]. Consequently, a decrease in LRP/LR, TERT and TRF-2 may allow the elevation of p53 and subsequent induction of apoptosis in cancerous cells (Figs. 1-3). Furthermore, as both LRP and TERT have been intricately linked to proliferation pathways like the MAPK pathway [38], knock-down of LRP and subsequently TERT and TRF-2, could severely impact the aggressiveness and rapid proliferation observed in cancer cells. Thus, by targeting LRP/LR with siRNA technology, its consequent effects on TERT/telomerase and TRF-2 observed in this study may compromise several key hallmarks of cancer.

The proteomics data obtained upon knock-down of LRP/LR in DLD-1 cells via siRNA technology identified several proteins that have novel interactions with LRP/LR, linking LRP/LR to other processes and thereby exposing novel roles for the receptor (Figs. 2 and 4). Additionally, when analysing the functions of each of these proteins, most were found to play roles such as: ribosomal processing and protein synthesis [3]; nucleus and chromatin maintenance [4]; cell proliferation and differentiation [39]; cell anchorage and cytoskeletal involvement [5] and lastly, apoptotic regulation [19] (See Tables S4-S12 for grouped proteins). In addition, other proteins identified were found to be involved in vesicle transportation and regulation as well as autophagy (Fig. 4). Novel stand-alone proteins were also found to be influenced by LRP/LR, which could provide additional information on how the receptor works. Observations from this technique suggest that several proteins involved in suppressing tumourigenesis were up-regulated, while proteins involved in promoting tumourigenesis were down-regulated. However, there have also been several proteins identified where their interactions with LRP/LR are not easily explained, highlighting the need for further experiments to confirm many of these interactions.

The MAPK pathway regulates various downstream molecules including kinases as well as transcription and translation factors [40]. In particular, one such protein is focal adhesion kinase (FAK) which, upon densitometric analysis, was shown to be down-regulated compared to non-transfected cells (Fig. 2a). FAK is a non-receptor tyrosine kinase found to play crucial roles in regulating cell proliferation, adhesion and survival in several cell types [41]. A previous study found that the LRP/LR-laminin-1 interaction allowed for FAK and LRP/LR to interact, thereby activating the MAPK signalling pathway and the anti-apoptotic Bcl-2 protein, ultimately leading to apoptotic inhibition [30]. Since FAK was down-regulated upon siRNA-mediated knock-down of LRP/LR in this study, this shows that these results are consistent with previous findings and confirms the relationship between FAK and LRP/LR [30]. In addition, it was also found that the knock-down of LRP/LR decreased the mRNA expression levels of Bcl-2, further confirming the results of the previous study (Fig. 3c) [30].

Another interesting protein linking LRP/LR to the MAPK pathway was the CREB (cyclic AMP-response element binding protein) that was found to be up-regulated (Figs. 2, 3a, b and d). This protein normally functions as a transcription factor that regulates various processes including cell growth and survival by binding to cAMP response elements (CRE) sequences [42]. CREB has also been implicated in genotoxic stressed-induced transcription as a result of DNA damage and oxidative stress [42]. For full transcriptional activity of the p53 tumour suppressor, the coactivator CBP/p300, is required to bind to p53 whereby the binding of these proteins is facilitated by phosphorylated CREB. Moreover, Chk-2-mediated phosphorylation (a protein also found to be significantly up-regulated upon LRP/LR knock-down) of p53 stabilizes the binding of CBP/p300 to p53 [43]. This results in p53 transactivating a number of proteins that may be used for apoptotic induction. The findings of the current study suggest that LRP/LR may link CREB, Chk-2 and p53, since a decrease in LRP/LR expression levels results in each of these proteins aiding in apoptotic induction.

Further analysis of the SWATH-MS data revealed that upon siRNA-mediated knock-down of LRP/LR, the AMP-activated protein kinase catalytic subunits α1 and 2 (AMPKα1 and 2) were both up-regulated, when compared to esiRNA-RLUC-transfected cells (Fig. 4). AMPK is known to primarily function in regulating energy and metabolism homeostasis [44]. The protein is activated upon stressors such as nutrient depletion and hypoxia, resulting in it also being a regulator of autophagy. Interestingly, a previous study revealed that CREB binds to AMPKα in order for the transactivation of p53 in response to energetic stress [44]. The authors therefore proposed that the CREB-AMPKα interaction facilitates the regulation of transcriptional p53. Taken together with the results from the current study where CREB was found to be up-regulated (Figs. 2 and 3d) upon knock-down of LRP/LR, it is suggested that the CREB-AMPKα association additionally facilitates p53-mediated apoptosis induced by the Human-RPSA siRNA.

SWATH-MS also demonstrated how the DLD-1 colorectal cancer cells responded to a decrease in the levels of the receptor, in that several enzymatic proteins involved in metabolism were also affected by LRP/LR down-regulation. This could most likely be due to the tumour cells adapting in response to stress – such as low nutrient levels, DNA damage and oxidative stress [45] – by undergoing autophagy. Cancer cells have been found to undergo autophagy to degrade damaged or unessential cell constituents. This degradation serves as an alternative energy source to sustain survival [45].

It is known that p53 primarily functions in the intrinsic apoptotic pathway however, there have been several studies showing that p53 is also able to play a central role in the extrinsic apoptotic pathway [46]. This is because p53 is able to activate the transcription of key proteins involved in both pathways including Bax of the intrinsic pathway and the Fas death receptor of the extrinsic pathway [46]. Moreover, cytochrome c release, which is normally an important incident in the intrinsic pathway, has also been found to occur in the extrinsic pathway [46]. From this study, the Apoptotic Proteome Profiler Array™ showed that cytochrome c was increased in the DLD-1 cells upon siRNA-mediated knock-down of LRP/LR, when compared to non-transfected cells (Fig. 2c). Moreover, we have previously found that DLD-1 cells also display significant increases in both caspase-8 and -9 upon siRNA-mediated knock-down of LRP/LR, when compared to non-transfected cells [47]. The results from this study show that upon siRNA-mediated knock-down of LRP/LR, Bax mRNA and protein expression levels are significantly increased (Figs. 2, 3c and b), correlating to the increased expression levels of p53 on account of genotoxic stress. The large increases of p53 and Bax may therefore have stimulated the increased release of cytochrome c, resulting in caspase-9 cleaving caspase-3 (Fig. 2c) – ultimately activating mitochondrial intrinsic apoptosis.

Another protein that confirms that siRNA-mediated knock-down of LRP/LR results in apoptosis is Histone H2AX that was found to be significantly up-regulated, when compared to esiRNA-RLUC-transfected cells (Table S5). H2AX is a protein marker that becomes phosphorylated to γH2AX in response to DNA damage and double stranded breaks which mostly accumulate due to dysfunctional telomeres [48]. Studies have shown that dysfunctional telomeres are able to activate a DNA damage response pathway involving γH2AX, ATM and p53 proteins, ultimately leading to cell cycle arrest and apoptosis of cancer cells [49]. In the current study, siRNA-mediated knock-down of LRP/LR not only significantly decreased hTERT expression levels and telomerase activity but also increased the expression of pro-apoptotic protein Bax and the p53 tumour suppressor. The fact that the expression of the DNA damage marker H2AX is also increased further reiterates that DLD-1 cells undergo apoptosis upon LRP/LR knock-down.

## Conclusions

From the current study, it is proposed that siRNA-mediated knock-down of LRP/LR induces cell death in the DLD-1 cells possibly as a result of diminished expression levels of telomerase activity, hTERT, and TRF-2. This consequently leads to the stabilisation and stimulation of p53 by the CREB-AMPKα interaction, resulting in the significant up-regulation of Bax as well as other pro-apoptotic proteins. In addition, FAK and β-catenin are down-regulated as well as several anti-apoptotic proteins. Subsequently, Bax promotes the release of cytochrome c, resulting in caspase-9 activation and apoptosis.

**Fig. 5:**
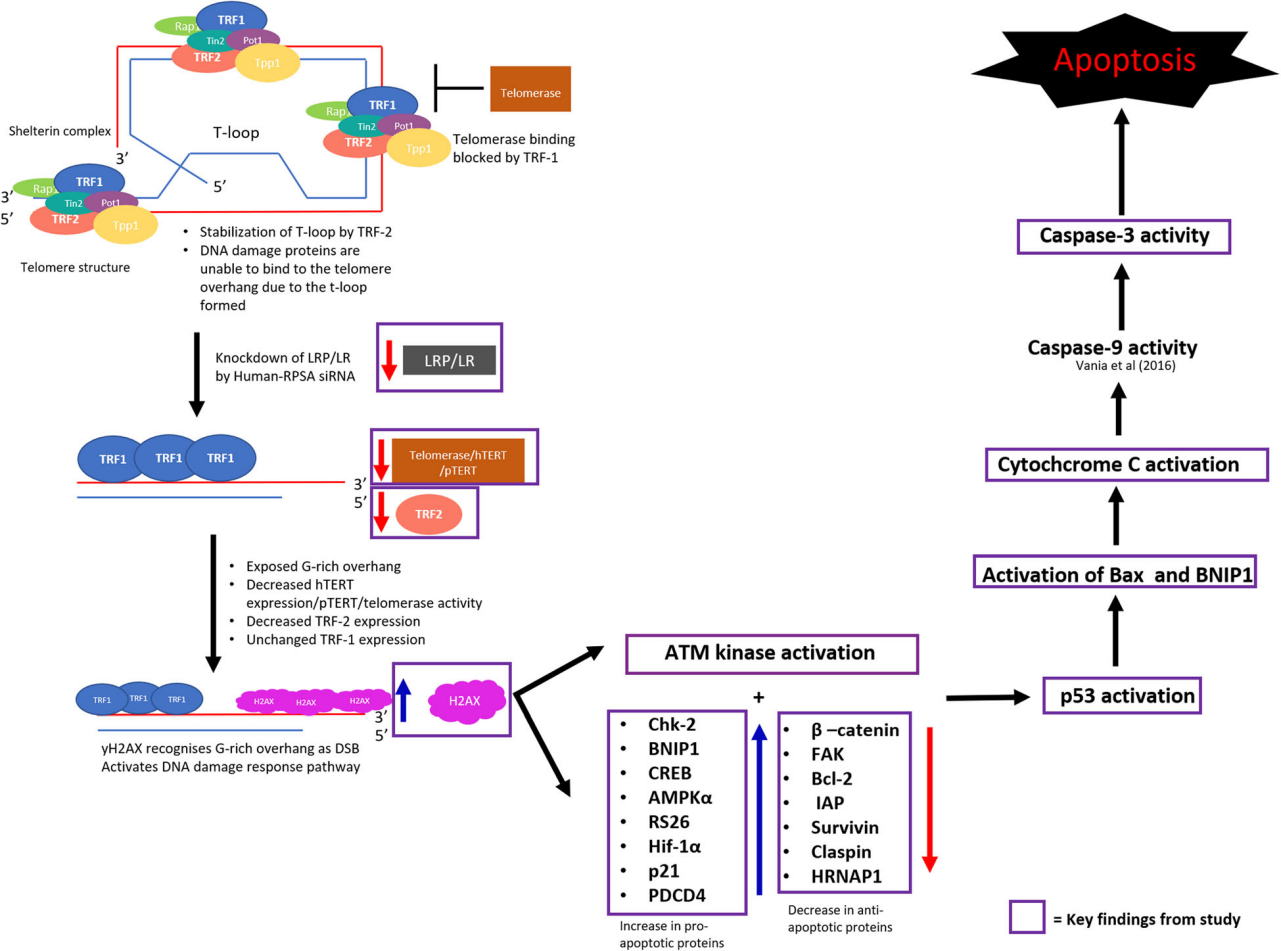
Graphical representation of the potential apoptotic pathway induced by the Human-RPSA siRNA in late (DLD-1) stage colorectal cancer cell. Under normal conditions, TRF-1 and TRF-2 binds to telomeres and negatively regulates telomere length by forming the t-loop structure at the ends of chromosomes to mask the G-rich overhang. Upon siRNA-mediated knock-down of LRP/LR, TRF-2 protein expression levels are decreased, leaving the ends of chromosomes exposed and free to fuse with each other, which can lead to genetic instability. The knock-down of LRP/LR was also found to decrease telomerase activity and hTERT expression levels, causing progressive telomere shortening and the inactivation of the telomere capping function, further contributing to chromosomal end fusion and genetic instability. This leads to the G-rich overhang being exposed and recognised as a double stranded break by γH2AX. This results in the activation of the DNA damage response pathway whereby ATM kinase activated p53. This leads to the activation of several other pro-apoptotic proteins as well as the inactivation of anti-apoptotic proteins, ultimately leading to apoptotic induction

Together, these findings show the important role that LRP/LR plays in maintaining cell viability and evading apoptosis. Moreover, the results reveal that siRNAs targeting LRP/LR as well as other proteins related to the receptor could be used as potential therapeutic tools for the treatment of late-stage colorectal cancer cells. Although interesting responses were observed in the colorectal cancer cells, the exact mechanism of action of LRP/LR in tumourigenesis is far from being fully understood. Figure 5 below shows a graphical representation of the possible apoptotic pathway induced by the Human-RPSA siRNA in DLD-1 cells.

## Supplementary Information

**Additional file 1.**

## Data Availability

All data generated or analysed during this study are included in this published article [and its supplementary information files].

## Abbreviations

AD: Alzheimer’s Disease
AMPK: AMP-activated protein kinase
APS: Ammonium persulfate
ATM: Ataxia-telangiectasia mutated
Bax: BCL2 associated X protein
BCA: Bicinchoninic acid
Bcl-2: B-cell lymphoma protein 2
BH3: Bcl-2 homology-3
BNIP: BCL2/adenovirus E1B 19 kDa interacting protein
BSA: Bovine serum albumin.
Caspases: Cysteine-dependent aspartate-specific proteases
CDK: Cyclin-dependent kinase
cDNA: Complementary DNA
CHAPS: 3-((3-cholamidopropyl) dimethylammonio)-1-propanesulfonate
CHK-2: Checkpoint kinase 2
cIAP1: Cellular inhibitor of apoptosis 1
CO_2_: Carbon dioxide
CREB: Cyclic AMP-response element binding protein
DDA: Data-independent analysis
DMSO: Dimethyl sulfoxide
DNA: Deoxyribonucleic acid
DTT: Dithiothreitol
EDTA: Ethylenediaminetetraacetic acid
ERK: Extracellular signal-regulated kinase
FACS: Fluorescence Activated Cell Scanning
FAK: Focal adhesion kinase
FCCC: Fox Chase Centre
FDR: False discovery rate
FITC: Fluorescein isothiocyanate
FBS: Fetal bovine serum
HCl: Hydrochloric acid
HRP: Horseradish peroxidase
HNRNPA1: Heterogeneous nuclear ribonucleoprotein A1
IAA: Imidazoleacetic acid
IAP: Inhibitor of apoptosis
LRP/LR: Laminin receptor precursor/high-affinity laminin receptor
MAP: Mitogen activated protein
mRNA: Messenger RNA
mtDNA: mitochondrial DNA
PAGE: Polyacrylamide gel electrophoresis
PBS: Phosphate buffered saline
PCA: Protocatechuic acid
PCD: Programmed cell death
PDCD4: Programmed Cell Death Protein 4
PI: Propidium iodide
PI3K: Phosphoinositide-3 kinase
POT1: Protection of telomeres-1
qPCR: Quantitative real-time PCR
RAP-1: Repressor activator protein-1
RNA: Ribonucleic acid
RNase: Ribonuclease
RPMI: Roswell Park Memorial Institute
RPSA: Ribosomal protein SA
RLUC: Renilla luciferase
SDS: Sodium dodecyl sulphate
siRNA: Small interfering RNA
SWATH-MS: Sequential Window Acquisition of All Theoretical Mass Spectra
TBE: Tris-Boric acid-EDTA
TEMED: Tetramethylethylenediamine
TERT: Telomerase reverse transcriptase
TRF-1/2: Telomeric repeat-binding factor-1/2
Tris: 2-Amino-2-(hydroxymethyl)-1,3-propandiol
TSE: Transmissible Spongiform Encephalopathies
XIAP: X-linked mammalian inhibitor of apoptosis protein
