# Supplementary material for: Knock-down of LRP/LR influences signalling pathways in late-stage colorectal carcinoma cells
Source: BMC Cancer. 2021 Apr 9;21:392. doi: 10.1186/s12885-021-08081-3 (PMC8035741; doi:10.1186/s12885-021-08081-3)
Supplement: Supplementary file 1 — Additional file 1. [file 12885_2021_8081_MOESM1_ESM.docx]

**Supplementary data section**

**Methods:**

**Cell cultivation:**

The DLD-1 cell line was cultured in Roswell Park Memorial Institute (RPMI) 1640 culture medium, supplemented with 10% (v/v) FBS, 1% (v/v) non-essential amino acids and 1% (v/v) Penicillin/Streptomycin in 75 cm^3^ culture flasks. Cells were sub-cultured twice a week by discarding the media and washing the cells in PBS to remove any residual media. This was followed by an incubation in 1 X Trypsin/EDTA at 37 °C with 5% CO2 in a humidified atmosphere for 5 minutes. The Trypsin/EDTA was inactivated with culture medium and cells were seeded at appropriate concentrations and dilution factors when necessary.

### Cell cycle analysis – Flow cytometry:

B)

A total of 100 000 DLD-1 colorectal cancer cells were seeded per well in 6-well tissue culture plates prior to transfection for 72 hours. The cells were then harvested and washed with 1 X PBS followed by a centrifugation at 420 x g for 10 minutes. Thereafter, cells were fixed in cold 70% ethanol in a dropwise manner while vortexing for fixation. Fixation took place for at least 24 hours at 4°C. Following this, cells were washed with 1 X PBS after which they were centrifuged twice at 420 x g for 10 minutes. The supernatant was discarded followed by the cells incubating in 200 µl of Guava® cell cycle reagent containing Propidium Iodide for 30 minutes in the dark. These resulting suspensions were evaluated using the BD Accuri C6 flow cytometer and software.

### Sequential Window Acquisition of All Theoretical Mass Spectra (SWATH MS):

#### *Sample clean-up and digestion*

All experiments were performed with a KingFisher™ Flex magnetic particle processing robot. The Fisher™ Flex system was configured for automated HILIC-protein clean-up and on-bead trypsin digest. Deep-well 96-plates were loaded in each carousel position with each plate filled as follows: 1) 96 well tip heads; 2) 10 µl, 20 mg/ml hyper porous magnetic HILIC micro spheres in 20% ethanol and 180 µl equilibration buffer (100 mM NH_4_Ac, 15% ACN pH 4.5); 3) equilibration Buffer (500 µl); 4) Protein extract mixed 1:1 with bind buffer (200 mM NH_4_Ac, 30% ACN pH 4.5), final volume of 100 µl; 5) 500 µl 95% ACN (wash 1); 6) 500 ul 95% ACN (wash 2); 8) 200 µl 50 mM ammonium formate pH 8.2 and Promega sequencing grade trypsin for an enzyme:protein ratio of 1:10. The Bindit programme was then run with the magnetic pins transferring the magnetic HILIC beads from position 2 to 8 and in the process binding proteins, washing off SDS and other contaminants and finally generating peptides ready for LC-MSMS analysis post the on-bead trypsin digest.

#### *Spectral library building (Data-dependent analysis)*

Post HILIC peptide samples were vacuum dried, resuspended in 2% ACN/0.2% FA and spiked with iRT peptide standards. Three injections were then performed per sample for each of the conditions. Analysis was performed using a Dionex Ultimate 3000 RSLC system coupled to an AB Sciex 6600 TipleTOF mass spectrometer. Peptides were first de-salted on an Acclaim PepMap C18 trap column (75 µm × 2 cm) for 5.5 min at 5 µl/min using 2% acetonitrile/0.2% formic acid, than separated on Acclain PepMap C18 nanoRSLC column (75 μm × 15 cm, 2 µm particle size) using a 60 min linear gradient at a flow-rate of 0.5 μl/min (A: 0.1% formic acid; B: 80% acetonitrile/0.1% formic acid). An electrospray voltage of 2.5 kV was applied to the fused silica emitter (New Objective: 20 μm ID x 5 cm, 10 μm tip). The 6600 TipleTOF mass spectrometer was operated in Data Dependant Acquisition mode. Precursor MS scans were acquired from *m/z* 400-1500 using an accumulation time of 250 ms followed by 80 MSMS scans, acquired from *m/z* 100-1800 at 25 ms each, for a total scan time of 2.3 sec. Multiply charge ions (2^+^ - 5^+^, 400 -1500 *m/z*) were automatically fragmented in Q2 collision cells using nitrogen as the collision gas.

#### *SWATH –MS analysis*

Three injections, representing each of the technical replicates, were performed per sample for each of the conditions. For SWATH–MS, the LC gradient used for spectral library building was applied. The SWATH-MS method consisted of the acquisition of 100 MS2 scans of overlapping sequential precursor isolation windows (variable m/z isolation width, 1 m/z overlap, high sensitivity mode) covering the 400 to 900 m/z mass range, as well as a single MS1 scan. The accumulation time was 300 ms for the MS1 scan and 25 ms for the MS2 scans for a total of 2.3 s total cycle time.

####

#### *Protein identification and spectral library building*

Raw data were searched against the human UNIPROT sequence database (reviewed entries, downloaded on 2 June 2017) supplemented with a list of common contaminating proteins as well as the sequences of the Biognosys iRT peptide retention time standards. Thereafter, data processing was performed using Protein Pilot (v 5.0.1). The following search settings were applied: trypsin as the proteolytic enzyme, IAA based alkylation, thorough search effort. False discovery rate (FDR) analysis was then performed with 1% global FDR cut-off applied at PSM, peptide and protein levels. A spectral library was constructed by importing the .group Protein Pilot output into the Skyline (v 4.1.1.18179) spectral library builder. A cut-off of 0.995, corresponding to 1% peptide FDR, was applied during import. The Biognosys iRT peptides were appended to the library in order to normalize the peptide retention time. The following filters were applied for peptide and protein import into Skyline: Tryptic peptide, size 5-36 amino acids, with up-to 1miss-cleavage and one matched cleavage site allowed; Structural modifications: Carbomedomethyl (Cys), Oxidation (Met), Acetylation (N-terminal); Precursor charge states 2-4, product charge states 1-2, product ions: b and y; Ion match tolerance of 0.1 m/z. Post protein, peptide and transition import into Skyline a decoy peptide list was generated by shuffling the sequences of all imported peptides.

####

#### *SWATH-MS processing*

SWATH data files were converted to mzML format as well as centroided using the Proteo Wizard MS Convert tool. The converted SWATH mzML files were imported into Skyline (v 4.1.1.18179). The following filters were applied for SWATH mzML import: Precursor charge states 2-4, product charge states 1-2, product ions: b and y; 3-6 transitions per peptide; Product m/z range 100-1800; MS1 filtering: Isotope peaks included (Count), Precursor mass analyzer (Centroid), Peaks (3), Mass accuracy (20 ppm); MS2 filtering: Acquisition method (DIA), Product mass analyzer (Centroid), Mass Accuracy (20 ppm) and an isolation scheme of 80 variable windows as per the SWATH method run in Analyst; For retention time filtering only scans within 10 min of the predicted iRT retention times were selected. Once all SWATH mzML files were imported all repeated peptides and proteins were removed and a peak scoring model was trained using mProphet and the decoy peptides generated during spectral library building. All peaks were then re-integrated using this model and only peptides with q-values of less than or equal to 0.01 were used for further processing.

**Sequences for siRNA used to down-regulate LRP/LR:**

**Table S1: Sequence of Human-RPSA, esiRNA-RPSA and control siRNA-RLUC used for down-regulation of LRP/LR**

| **siRNA** | **Organism** | **Sequence** | **Transfection reagent** |
| --- | --- | --- | --- |
| ON-TARGETplus SMARTpool Human-RPSA | Homo sapiens | 4 RPSA siRNA pooled together (targets 4 regions of LRP/LR):  Target sequence 1:  CGACAUGAGUUGUACUUCU  Target sequence 2:  GAUUGCAUAUCAAAGCAUA  Target sequence 3:  GGUCAUGCCUGAUCUGUAC  Target sequence 4:  UAUCAUAAAUCUCAAGAGG | DharmaFect1 |
| esiRNA-RPSA | Homo sapiens | CCTCTCACGGAGGCATCTTATGTTAACCTACCTACCATTGCGC  TGTGTAACACAGATTCTCCTCTGCGCTATGTGGACATTGCCAT  CCCATGCAACAACAAGGGAGCTCACTCAGTGGGTTTGATGTGG  TGGATGCTGGCTCGGGAAGTTCTGCGCATGCGTGGCACCATTT  CCCGTGAACACCCATGGGAGGTCATGCCTGATCTGTACTTCTA  CAGAGATCCTGAAGAGATTGAAAAAGAAGAGCAGGCTGCTGCT  GAGAAGGCAGTGACCAAGGAGGAATTTCAGGGTGAATGGACT  GCTCCCGCTCCTGAGTTCACTGCTACTCAGCCTGAGGTTGCAG  ACTGGTCTGAAGGTGTACAGGTGCCCTCTGTGCCTATTCAGCA  ATTCCCTACTGAAGACTGGAGCG | Mission transfection reagent |
| esiRNA-RLUC | Homo sapiens | GATAACTGGTCCGCAGTGGTGGGCCAGATGTAAACAAATGAAT  GTTCTTGATTCATTTATTAATTATTATGATTCAGAAAAACATGC  AGAAAATGCTGTTATTTTTTTACATGGTAACGCGGCCTCTTCT  TATTTATGGCGACATGTTGTGCCACATATTGAGCCAGTAGCGC  GGTGTATTATACCAGACCTTATTGGTATGGGCAAATCAGGCAA  ATCTGGTAATGGTTCTTATAGGTTACTTGATCATTACAAATAT  CTTACTGCATGGTTTGAACTTCTTAATTTACCAAAGAAGATCAT  TTTTGTCGGCCATGATTGGGGTGCTTGTTTGGCATTTCATTAT  AGCTATGAGCATCAAGATAAGATCAAAGCAATAGTTCACGCTG  AAAGTGTAGTAGATGTGATTGAATCATGGGATGAATGG | Mission transfection reagent |

**Transfection procedure for Human-RPSA:**

Lyophilized Human-RPSA (5nmol/20nmol) was reconstituted in 100µl or 250μl of 1X RNase free siRNA buffer, respectively, before use in order to make a 20 μM stock. The table below shows amounts of siRNA and corresponding components used for transfections of a 6-well plate and 24-well plate.

**Table S2: Transfection components and volumes for Human-RPSA**

| **Reagent** | **6-well plate (Total volume of 2 ml per plate) (μl)** | **24-well plate (Total volume of 500 µl per plate) (μl)** |
| --- | --- | --- |
| Opti-MEM media – for addition of siRNA | 190 | 47.5 |
| Human-RPSA | 10 | 2.5 |
| Opti-MEM media – for addition of transfection reagent | 190 | 47.5 |
| DharmaFect transfection reagent | 10 | 2.5 |

**Transfection procedure:**

Reconstituted Human-RPSA was added to the corresponding volume of Opti-MEM media in a micro centrifuge tube. In a second tube, transfection reagent was added to the corresponding volume of Opti-MEM media. Both tubes were incubated for 5 minutes at room temperature and mixed together. The resultant siRNA solution was further incubated for 20 minutes at room temperature. The suggested volumes of culture media were added into the 6-or 24-well plates containing the seeded cells and thereafter the resultant siRNA solution was added to the cells.

**Transfection procedure for esiRNA-RPSA and esiRNA-RLUC:**

esiRNA-RPSA (200 ng /µl) and esiRNA-RLUC (200 ng /µl) are purchased reconstituted. The table below shows volumes of esiRNA and corresponding components used for transfections of a 6-well plate and 24-well plate:

**Table S3: Transfection components and volumes for esiRNA-RLUC**

| **Reagent** | **6-well plate (Total volume of 2 ml per plate) (μl)** | **24-well plate (Total volume of 500 µl per plate) (μl)** |
| --- | --- | --- |
| Opti-MEM media – for addition of esiRNA | 250 | 50 |
| esiRNA-RPSA or esiRNA-RLUC | 5 | 1.5 |
| Opti-MEM media – for addition of transfection reagent | 250 | 50 |
| Mission transfection reagent | 5 | 1.5 |

**Transfection procedure:**

esiRNA-RPSA or esiRNA-RLUC was added to the corresponding volume of Opti-MEM media in a micro centrifuge tube. In a second tube, transfection reagent was added to the corresponding volume of Opti-MEM media. Both tubes were incubated for 5 minutes at room temperature and the contents of both tubes were mixed together. The suggested volumes of culture media were added into the 6-or 24-well plates containing the seeded cells and thereafter the resultant siRNA solution was added to the cells.

### siRNA confirmation:

### siRNA technology successfully results in knock-down of LRP expression in late stage colorectal carcinoma cells.

To understand the effect LRP/LR expression has on early and late stage colorectal cancer cell viability, down-regulation of the receptor was performed. Once the late (DLD-1) stage colorectal carcinoma cells were transfected with the Dharmacon™ ON-TARGETplus SMARTpool Human-RPSA siRNA (targets four different regions of the 37kDa LRP mRNA), evaluation of LRP levels was performed via western blotting and quantitative real-time PCR. To visualize whether the cells were undergoing morphological changes, bright field microscopy images of the DLD-1 cells were taken before and after Human-RPSA siRNA transfection of 72 hours. It was found that the upon transfection with Human-RPSA siRNA, the DLD-1 cells appeared to be reduced in size with diminished cell membranes as well as condensed nuclei – all indicative of apoptotic induction (Fig. S1). To confirm this, Western blotting was performed, and analysis showed that 37 kDa LRP was significantly knocked down in DLD-1 cells when transfected with the Human-RPSA siRNA. The DLD-1 transfected cells exhibited a 78% decrease in 37 kDa LRP expression, when compared to cells that were not transfected (Fig.S2). Additionally, treating DLD-1 cells with the negative control siRNA, MISSION® esiRNA-RLUC, indicated no notable change in 37 kDa LRP expression levels in comparison to cells that were not transfected (Fig. S2). In addition, it was found that when DLD-1 cells were transfected with Human-RPSA siRNA, the relative mRNA expression levels of LRP/LR were significantly decreased by 0.9-fold, further confirming that Human-RPSA siRNA is successful in down-regulating the receptor as seen in Figure S3.


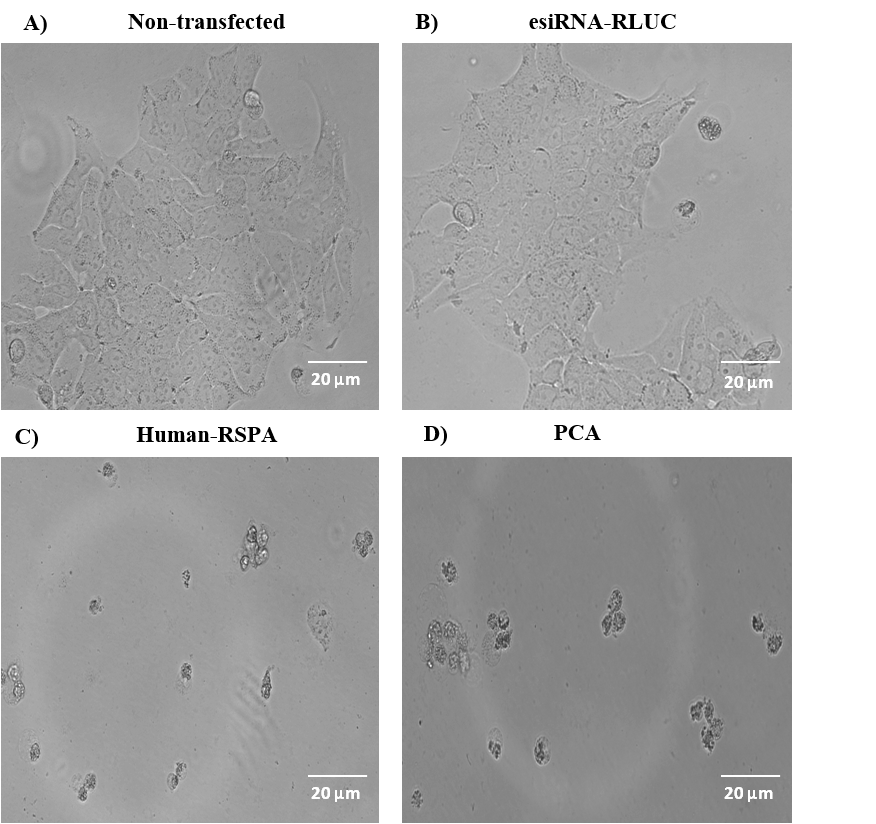


**Figure S1: Late stage (DLD-1) cells observed using bright field microscopy. A) and B)** Non-transfected and esiRNA-RLUC (negative control) transfected cells are found to be large with uncompromised membrane integrity. **C) and B)** Human-RPSA-transfected and PCA (positive control) treated cells are found to have a reduced size together with compromised membrane integrity i.e. membrane blebbing and condensed nuclei – indicative of apoptosis occurring. Images were obtained at 200X magnification. Scale bars are indicative of 20 µm (Adopted from Vania et al, 2018 [1].


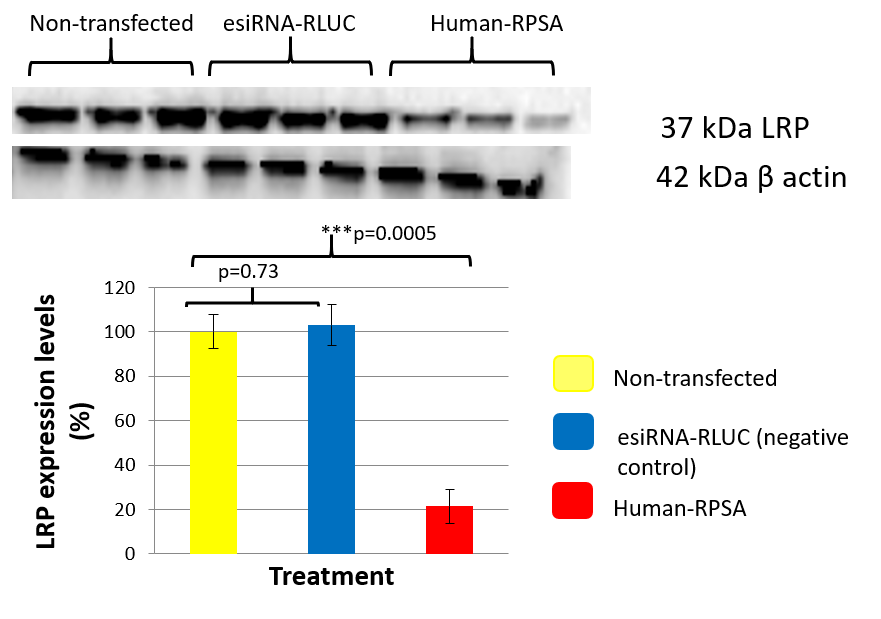


Figure S2: The effect of siRNA-mediated knock-down on LRP expression in late (DLD-1) stage colorectal cancer cells**.** Upon transfection of SW-480 (A) and DLD-1 (B) cells with Human-RPSA siRNA, a significant 79% decrease in LRP expression levels was revealed, in contrast to cells that were not transfected. Densitometric analysis of LRP levels was performed where levels of the non-transfected cells were set to 100%. β-actin was used as the loading control and the graphs are representative of an average of experiments performed in triplicate. *p < 0.05, **p < 0.01, ***p < 0.001, significant: The One-way ANOVA was significant and a Bonferroni corrected post-hoc *t*-test indicated that the Human-RPSA samples are significantly lower than the non-transfected and esiRNA-RLUC samples (Adapted from Vania et al, 2018 [1]).


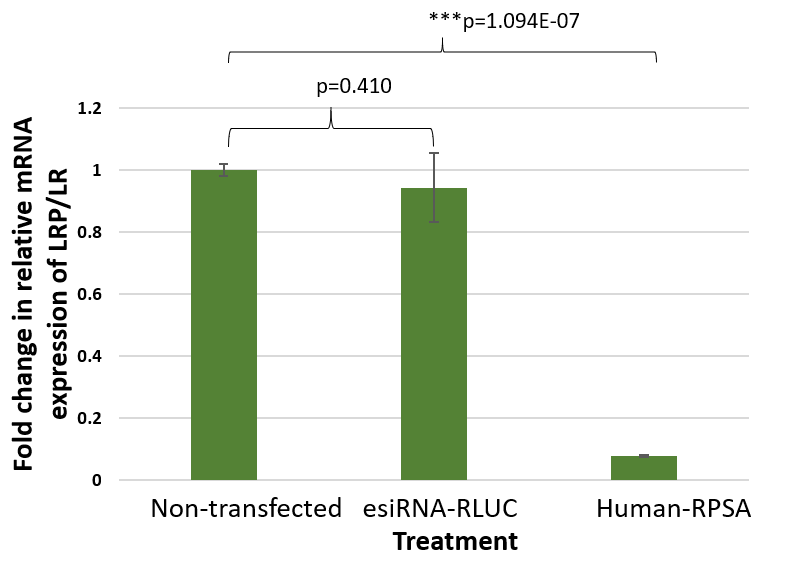


Figure S3: Relative mRNA expression levels of LRP/LR in late (DLD-1) stage colorectal cancer cells**.** Upon transfection with the Human-RPSA siRNA, relative LRP/LR mRNA expression levels decreased by 0.9-fold, when compared to non-transfected cells, confirming that the siRNA successfully down-regulates LRP/LR. esiRNA-RLUC served as the negative control. This data represents three biological replicates with two technical repeats with newly transfected samples each time. *p < 0.05, **p < 0.01, ***p < 0.001, significant: The One-way ANOVA was significant, and a Bonferroni corrected post-hoc *t*-test indicated that the Human-RPSA samples are significantly lower than the non-transfected and esiRNA-RLUC samples.

**Cell cycle analysis:**

### siRNA-mediated knock-down of LRP/LR expression causes apoptotic cell cycle arrest in late stage colorectal cancer cells.

Due to LRP/LR’s role in cell viability maintenance, cell cycle analysis was performed to not only obtain insight into the effect of LRP/LR knockdown but to also confirm the occurrence of apoptosis, since disruption of the cell cycle leads to the development of cancer. DLD-1 cells were transfected with the Human-RPSA siRNA for a duration of 3 days as well as 5 days in order to downregulate LRP/LR.

Upon Human-RPSA transfection of DLD-1 cells for 3-days, there was a significant 15% increase in the number of dead cells in the sub G0/G1 stage (apoptotic stage) and 17.4% decrease in G0/G1 phase, indicating the occurrence of apoptosis (Fig.S4). Although this result was significant, it was not a substantial increase. It is known that apoptosis is a time-dependent process where the occurrence of apoptotic biochemical alterations to cells may depend on several internal cellular factors, where some alterations may take longer than others to show an effect [2]. Thus, it was decided to extend the transfection period by 48 hours, for a total of 5 days to ensure that the period of apoptotic activity was not missed. Upon transfection of DLD-1 cells with Human-RPSA for 5 days, there was a significant 41% increase in the number of dead cells in the sub G0/G1 stage (apoptotic stage) with a 43% decrease in G0/G1 phase, indicating a higher percentage of cells undergoing apoptosis (Fig. S4b). It its noteworthy that there was no cell cycle arrest in any other particular phase of the cell cycle. esiRNA-RLUC and PCA acted as the negative and positive controls, respectively.


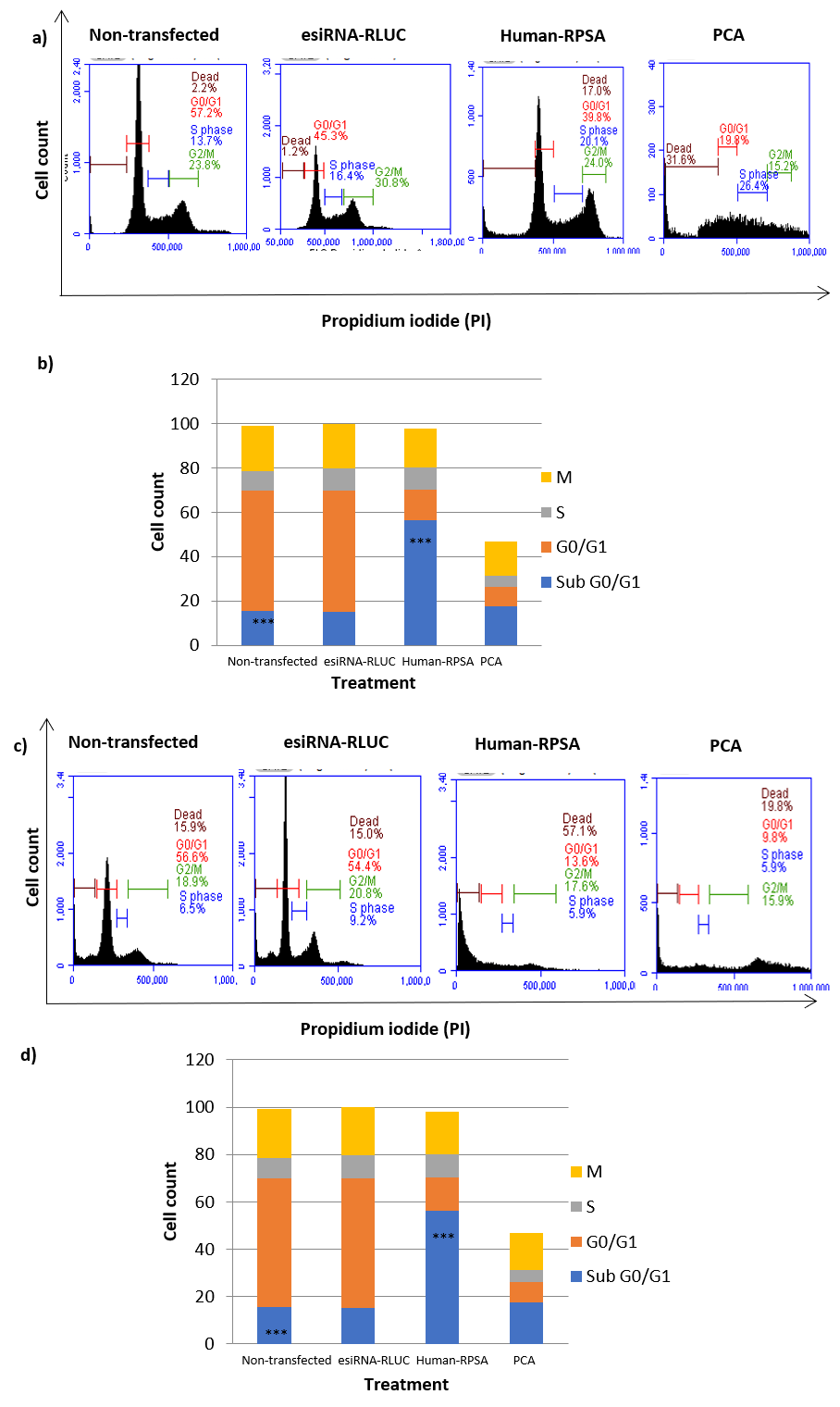


Figure S4: Cell cycle analysis on late (DLD-1) stage colorectal cancer cells after 3- and 5-day transfections with Human-RPSA siRNA**. (a)** Most non-transfected cells are in the G0/G1 stage of the cell cycle with only 2.2% cells found dead. Upon transfection with Human-RPSA siRNA, there is a 15% increase in the number of dead cells in the sub G0/G1 stage (apoptotic stage) and a 17.4% decrease in G0/G1 phase, indicating the occurrence of apoptosis. esiRNA-RLUC and PCA acted as the negative and positive controls, respectively. **(b)** It can be seen that there is a significant increase in the sub G0/G1 phase in cells transfected with Human-RPSA siRNA. ***p=0.0008, this data represents three biological replicates which were completed in triplicate. **c)** Most non-transfected cells are in the G0/G1 stage of the cell cycle with only 15.9% cells found dead. Upon transfection with Human-RPSA siRNA, there is a 41% increase in the number of dead cells in the sub G0/G1 stage (apoptotic stage) while there was a 43% decrease in G0/G1 phase, indicating the occurrence of apoptosis. esiRNA-RLUC and PCA acted as the negative and positive controls, respectively. (**d)** It can be seen that there is a significant increase in the sub G0/G1 phase in cells transfected with Human-RPSA. ***p=0.0003, this data represents three biological replicates which were completed in triplicate. *p < 0.05, **p < 0.01, ***p < 0.001; One-way ANOVA and two-tailed students *t*-test with Bonferroni post hoc analysis.

**Uncropped/full-length blots for Figure 1 of manuscript:**

A pre-stained molecular weight marker was used to view all gels. The molecular weight marker was viewed on all gels before transferring to membranes for western blotting.


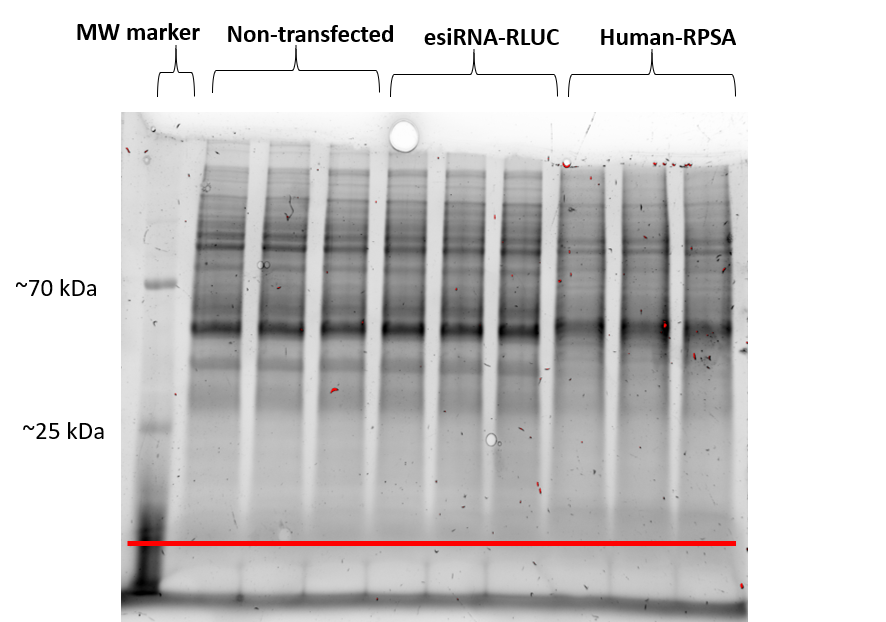


**Figure S5: Uncropped reference gel of DLD-1 colorectal protein samples. Proteins were separated at 120 V for 1 h 30 min until the loading dye reached the bottom (shown in red).**


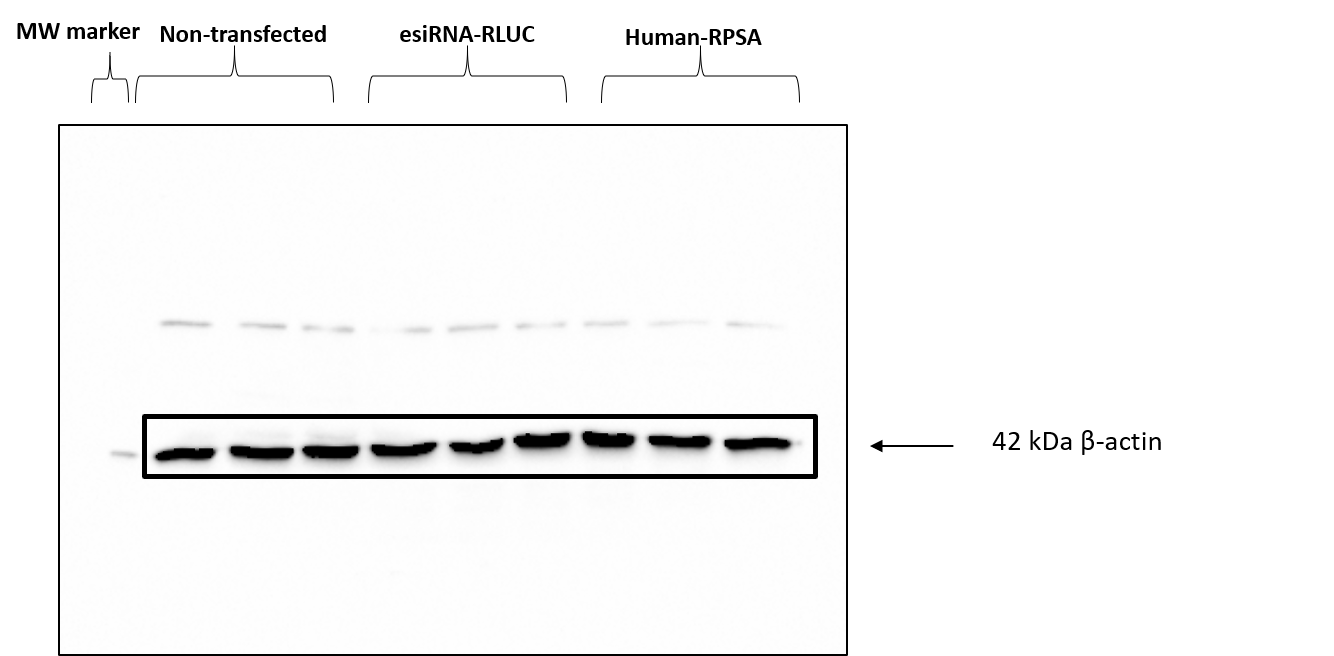


**Figure S6: Uncropped blot of β-actin (loading control) in DLD-1 late stage colorectal cancer cells.**


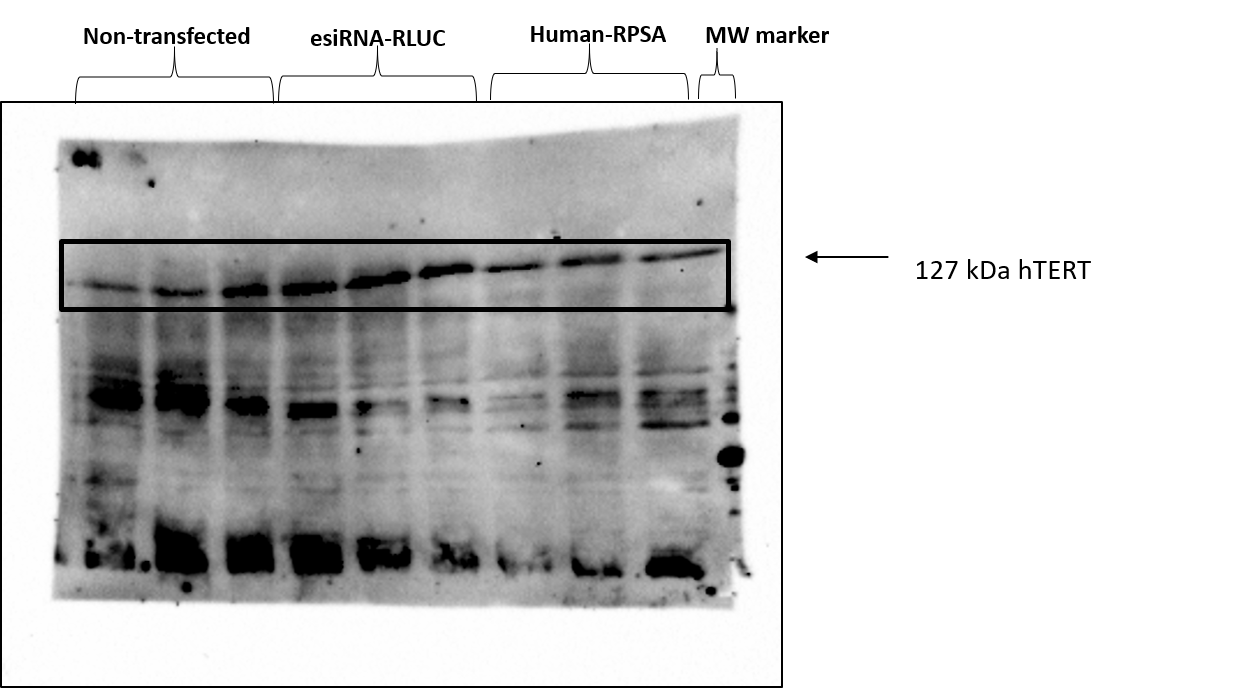


**Figure S7: Uncropped blot of hTERT protein upon** siRNA-mediated knock-down of LRP/LR in DLD-1 late stage colorectal cancer cells.


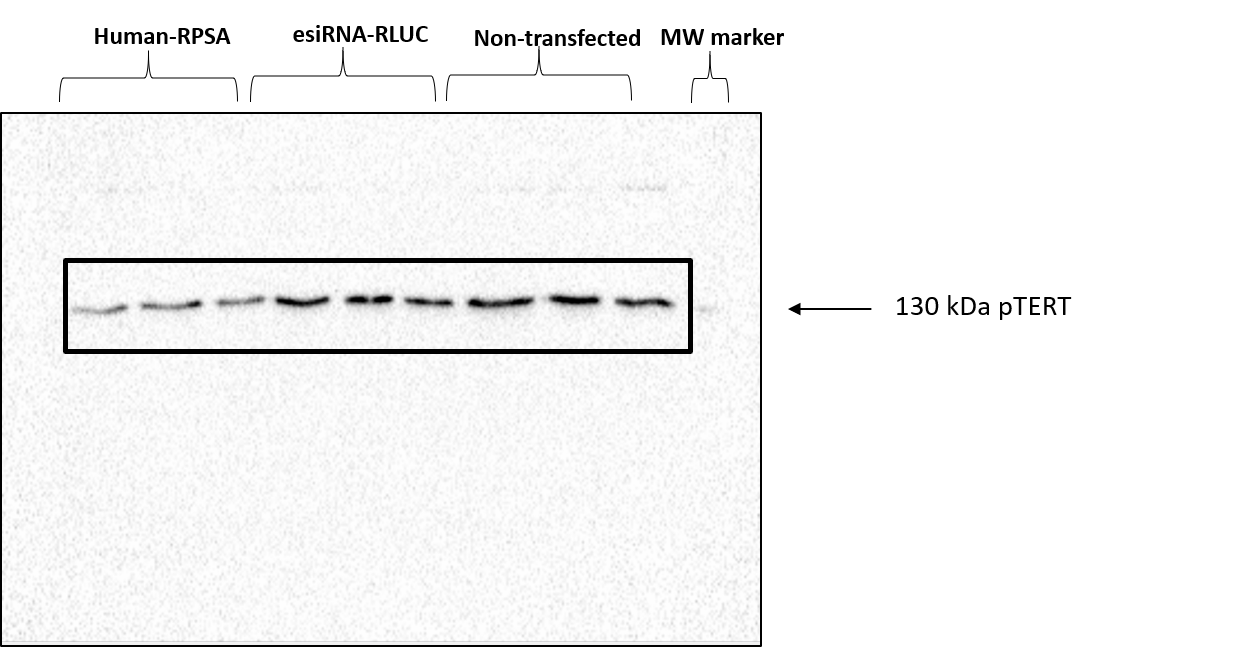


**Figure S8: Uncropped blot of pTERT protein upon** siRNA-mediated knock-down of LRP/LR in DLD-1 late stage colorectal cancer cells.


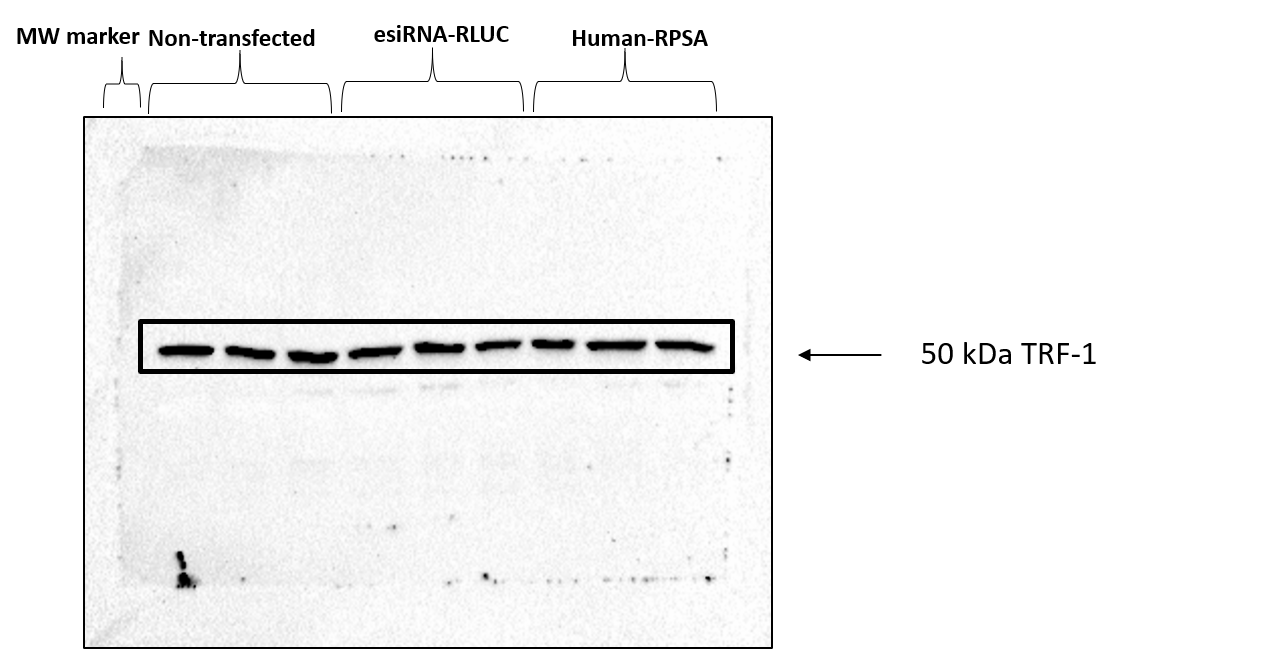


**Figure S9: Uncropped blot of TRF-1 protein upon** siRNA-mediated knock-down of LRP/LR in DLD-1 late stage colorectal cancer cells.


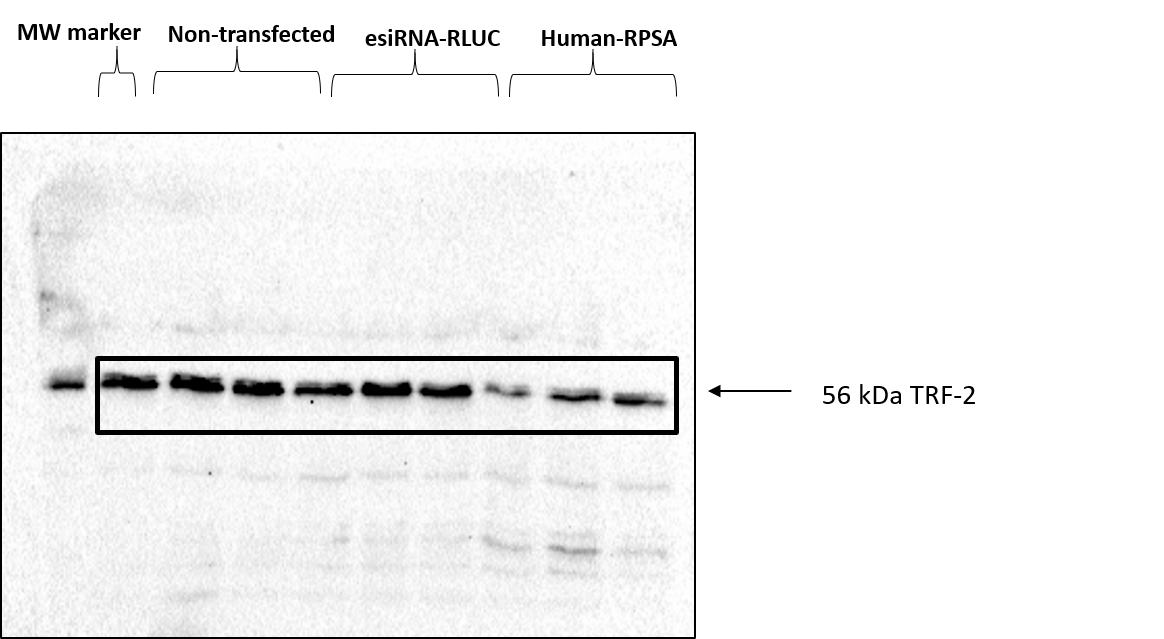


**Figure S10: Uncropped blot of TRF-2 protein upon** siRNA-mediated knock-down of LRP/LR in DLD-1 late stage colorectal cancer cells.

**Western blot performed prior to Proteome Profiler Antibody Arrays:**


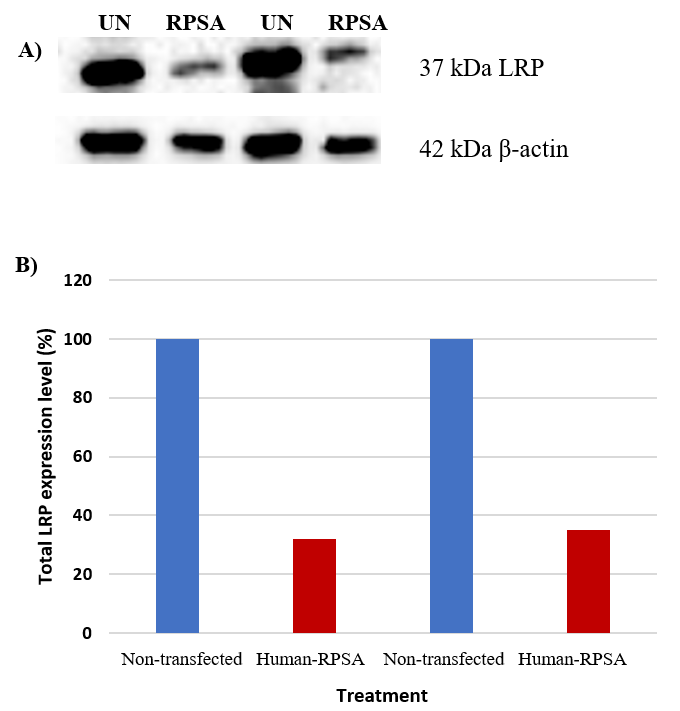


**Figure S11: Representative western blot of LRP/LR levels prior to performing Proteome Profiler Antibody Arrays.** Densitometric analysis revealed that upon transfection with Human-RPSA, LRP/LR was down-regulated by 70% when compared to non-transfected cells. All experiments for each array were performed using lysates that had 70% LRP/LR down-regulation for comparisons to take place.

**Visual representation of Proteome Profiler Arrays™ with corresponding coordinates:**


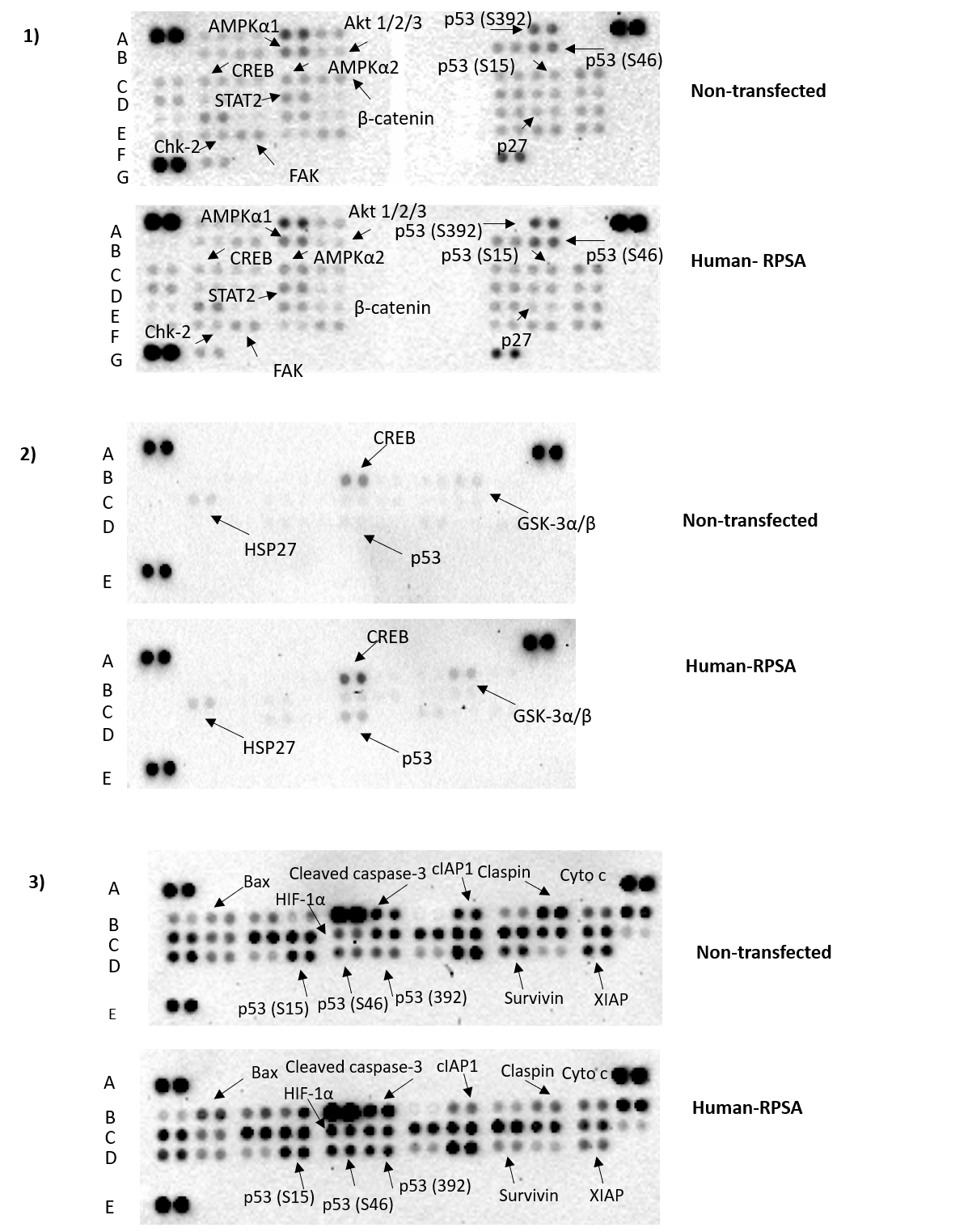


**Figure S12: Human Phospho-MAPK, MAPK and Apoptotic Proteome Profiler Array™ membranes. 1)** Non-transfected and Human-RPSA transfected DLD-1 cell lysates were incubated on the Human Phospho-MAPK Proteome Profiler Antibody membrane arrays. **2)** Non-transfected and Human-RPSA transfected DLD-1 cell lysates were incubated on the Human MAPK Proteome Profiler Antibody membrane arrays. When LRP/LR is down-regulated with Human RPSA siRNA, CREB and p53 proteins are found to be visible increased. **3)** Non-transfected and transfected DLD-1 cell lysates were incubated on the Human Apoptotic Proteome Profiler Antibody membrane arrays. A visible increase in several apoptotic proteins upon Human-RPSA siRNA transfection in comparison to the non-transfected cells was observed. Signals for each protein are displayed as a pair of spots, with three pairs of dark reference spots on the lower left, upper right and upper left corners for alignment. PBS spots (designated regions that had consistent background with no capture sites) were used as the negative control where each value was subtracted from PBS and divided by its corresponding reference spot.


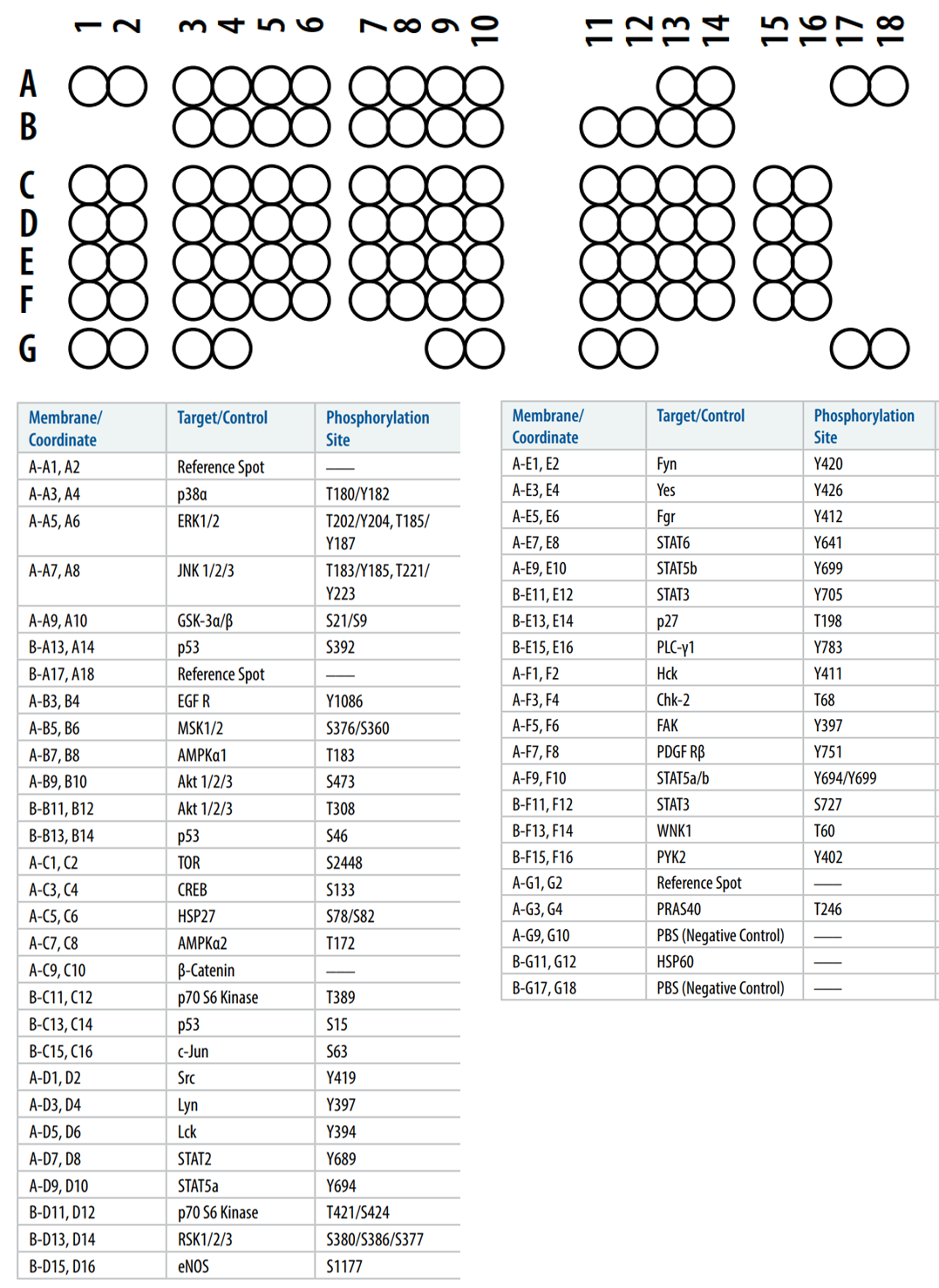


**Figure S13: Schematic of human Phospho-MAPK Proteome Profiler Antibody Array™ with corresponding coordinates.** Visual representation of the membrane array consisting of several proteins with their corresponding coordinates (shown below array). These coordinates are used to locate each protein on the array for analyte identification (Image obtained from phospho-MAPK array kit).


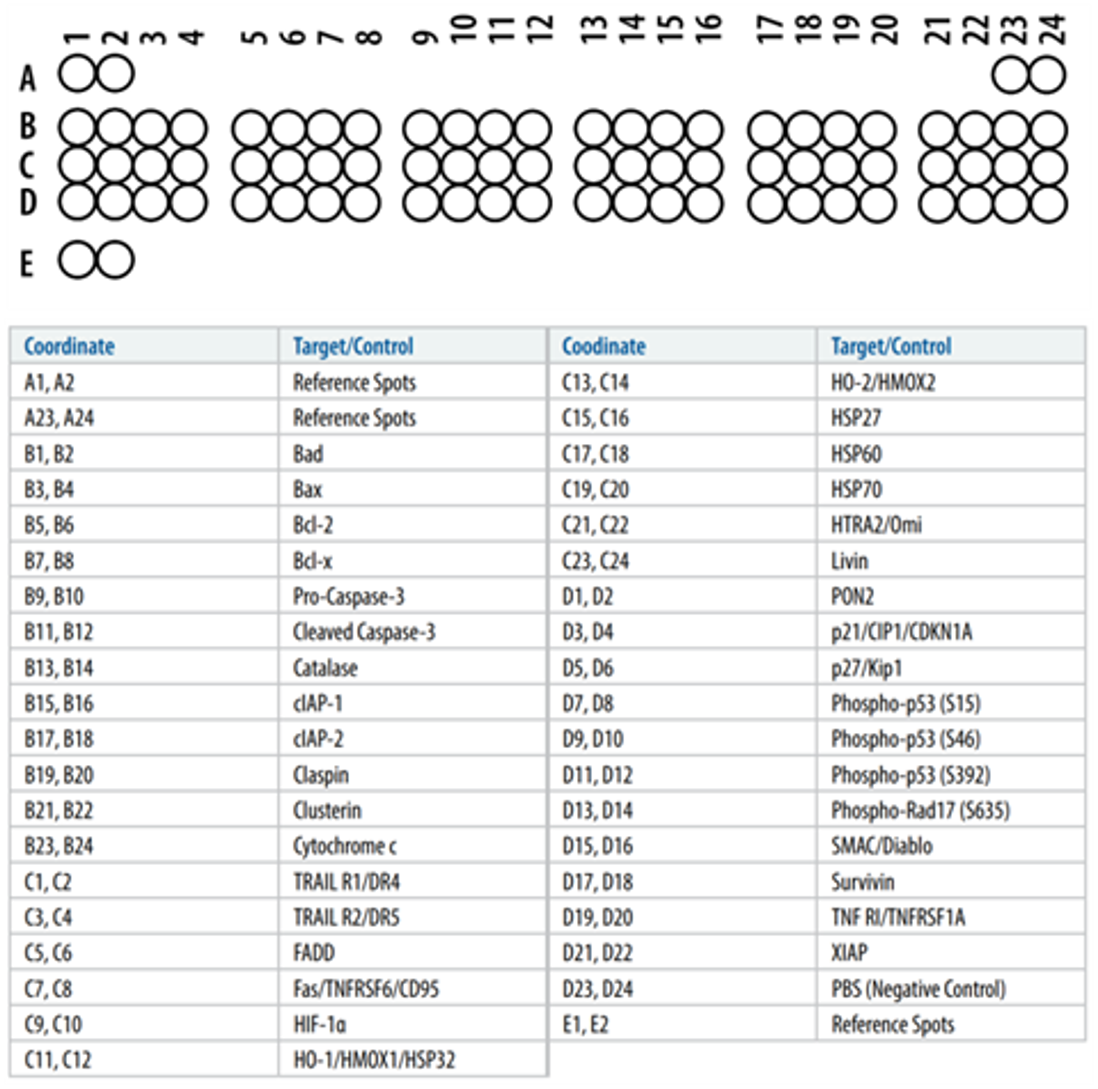


**Figure S14: Schematic of human Apoptosis Proteome Profiler Antibody Array™ with corresponding coordinates.** Visual representation of the membrane array consisting of several proteins with corresponding coordinates (shown below array). These coordinates are used to locate each protein on the array for analyte identification (Image obtained from Apoptosis array kit).

**Proteins obtained from SWATH-MS analysis:**

Table S4: List of down-regulated proteins with significant fold changes of two and above observed upon siRNA-mediated knock-down of LRP/LR.

| **Fold change** | **Protein** | **Name** | **P- value** | **Function** |
| --- | --- | --- | --- | --- |
| -8.20 | sp\|P09651\| HnRNPA1_HUMAN | Heterogeneous nuclear ribonucleoprotein A1 | 0.027 | Pre-mRNA processing, mRNA transport and metabolism [3, 4] |
| -5.75 | sp\|Q14244\|MAP7_HUMAN | Ensconsin | 0.001 | Microtubule stabilizing protein, involved in cellular differentiation and motility [5, 6] |
| -5.62 | sp\|O96028\|NSD2_HUMAN | Histone-lysine N-methyltransferase | 0.043 | Transcription regulator [7] |
| -5.04 | sp\|Q6QEF8\|CORO6_HUMAN | Coronin-like protein E | 0.009 | Actin binding protein, interaction with microtubules [8] |
| -4.95 | sp\|Q9UKY7\|CDV3_HUMAN | Carnitine deficiency-associated gene expressed in ventricle 3 | 0.001 | Biomarker for Hepatocellular carcinoma. Involved in cell proliferation [9] |
| -4.71 | sp\|Q9HD42\|CHM1A_HUMAN | Charged Multivesicular Body | 0.032 | Multivesicular body sorting of proteins to the interiors of lysosomes [10] |
| -4.64 | sp\|P17152\|TMM11_HUMAN | Transmembrane protein 11 | 0.038 | Mitochondrial morphogenesis [11] |
| -4.63 | sp\|Q9NYF8\|BCLF1_HUMAN | Bcl-2-associated transcription factor 1 | 0.040 | Apoptotic induction [12] |
| -4.48 | sp\|Q9H832\|UBE2Z_HUMAN | Ubiquitin conjugating enzyme E2 Z | 0.033 | Proteolysis [13] |
| -4.43 | sp\|Q7L9L4\|MOB1B_HUMAN | Mps one binder kinase activator-like 1A | 0.017 | Protein kinase essential for spindle pole body duplication and mitotic checkpoint regulation/ tumour suppressor properties [14] |
| -4.39 | sp\|Q9UK76\|JUPI1_HUMAN | Jupiter Microtubule Associated Homolog 1 | 0.011 | Cell cycle and cell adhesion regulation |
| -4.28 | sp\|P41567\|EIF1_HUMAN | Eukaryotic translation initiation factor 1 | 0.022 | Initiation of mRNA translation [15] |
| -4.26 | sp\|O14530\|TXND9_HUMAN | Thioredoxin Domain Containing 9 | 0.016 | Cell differentiation/ transcription [16] |
| -4.02 | sp\|P41222\|PTGDS_HUMAN | Prostaglandin-H2 D-isomerase | 0.013 | Cancer cell suppression [17] |
| -4.01 | sp\|Q14118\|DAG1_HUMAN | Dystroglycan | 0.048 | Receptor for laminin; involved in basement membrane assembly and cell survival [18] |
| -3.94 | sp\|Q9NWW5\|CLN6_HUMAN | Ceroid-lipofuscinosis neuronal protein 6 | 0.046 | Regulates the transportation from the ER to lysosomes [19] |
| -3.50 | sp\|Q7Z478\|DHX29_HUMAN | DExH-box helicase 29 | 0.008 | Initiation of mRNA translation [20] |
| -3.45 | sp\|Q9UJC5\|SH3L2_HUMAN | SH3 domain-binding glutamic acid-rich-like protein | 0.042 | Reduction of many intra-cellular protein disulphides [21] |
| -3.39 | sp\|P20042\|IF2B_HUMAN | Eukaryotic translation initiation factor 2 subunit 2 | 0.027 | Translation initiation factor activity [15] |
| -3.39 | sp\|P15529\|MCP_HUMAN | Monocyte chemotactic protein | 0.011 | Monocyte activation [22] |
| -3.32 | sp\|Q9UKI8\|TLK1_HUMAN | Serine/threonine-protein kinase tousled-like 1 | 0.033 | chromatin assembly, DNA repair, transcription, and chromosome segregation [23] |
| -3.29 | sp\|Q8N806\|UBR7_HUMAN | Putative E3 ubiquitin-protein ligase | 0.006 | Pathway protein ubiquitination [24] |
| -3.12 | sp\|P15151\|PVR_HUMAN | Poliovirus receptor/ PVR Cell Adhesion Molecule | 0.006 | Mediates cell adhesion, tumour cell invasion and migration [25] |
| -2.95 | sp\|P02751\|FINC_HUMAN | Fibronectin 1 | 0.0128 | Cell adhesion, differentiation, wound healing and migration [26] |
| -2.92 | sp\|Q9Y639\|NPTN_HUMAN | Neuroplastin | 0.035 | Cell-cell interactions or cell-substrate interactions [27] |
| -2.91 | sp\|P62875\|RPAB5_HUMAN | DNA-directed RNA polymerases I, II, and III subunit | 0.002 | Component of RNA polymerases I, II and III which synthesize ribosomal RNA precursors, mRNA precursors /transcription and DNA binding [28] |
| -2.85 | sp\|P54105\|ICLN_HUMAN | Methylosome subunit pICln | 0.041 | Splicing cellular pre-mRNAs [29] |
| -2.79 | sp\|Q06481\|APLP2_HUMAN | Amyloid-like protein 2 | 0.049 | Important modulator of glucose and insulin homeostasis; plays a role in cell proliferation and migration [30] |
| -2.79 | sp\|O60610\|DIAP1_HUMAN | Death-associated inhibitor of apoptosis 1 | 0.013 | Anti-apoptotic regulator [31] |
| -2.62 | sp\|Q8TAT6\|NPL4_HUMAN | Nuclear protein localization protein 4 | 0.034 | Endoplasmic reticulum-associated degradation; regulates ubiquitin-mediated mitochondria protein degradation [32] |
| -2.53 | sp\|Q99439\|CNN2_HUMAN | Calponin-2 | 0.048 | Actin filament-associated regulatory protein, involved in the regulation and modulation of smooth muscle contraction/cell proliferation, cell migration, and platelet adhesion [33] |
| -2.52 | sp\|Q15370\|ELOB_HUMAN | Elongin B | 0.047 | Transcription elongation factor activity [34] |
| -2.50 | sp\|P61916\|NPC2_HUMAN | Intracellular cholesterol transporter 2 | 0.011 | Cholesterol regulation [35] |
| -2.44 | sp\|O15405\|TOX3_HUMAN | TOX high mobility group box family member 3 | 0.005 | Transcription factor [36] |
| -2.43 | sp\|O96005\|CLPT1_HUMAN | ATP-dependent Clp protease ATP-binding subunit | 0.024 | Regulates the assembly of the Clp protease system [37] |
| -2.40 | sp\|Q13546\|RIPK1_HUMAN | Receptor-interacting serine/threonine-protein kinase 1 | 0.040 | Plays a role in apoptosis and necroptosis induction [38] |
| -2.37 | sp\|Q86UK7\|ZN598_HUMAN | Zinc Finger Protein 598 | 0.043 | Cell proliferation, differentiation, and apoptosis |
| -2.36 | sp\|P63220\|RS21_HUMAN | Ribosomal Protein S21 | 0.016 | Ribosomal protein that is a component of the 40S subunit [39] |
| -2.17 | sp\|Q15417\|CNN3_HUMAN | Calponin-3 | 0.040 | Regulates and modulates smooth muscle contraction/actin binding and calmodulin binding [33] |
| -2.12 | sp\|P07203\|GPX1_HUMAN | Glutathione peroxidase 1 | 0.012 | Protects cells from oxidative stress [40] |
| -2.06 | sp\|Q5JRA6\|TGO1_HUMAN | Transport and Golgi organization protein 1/Melanoma Inhibitory Activity Protein 3 | 0.023 | Transport of cargos that are too large to fit into COPII-coated vesicles from the endoplasmic reticulum / promotes the formation of metastases by inhibiting the attachment of melanoma cells to the extracellular matrix [41] |

Table S5: List of Up-regulated proteins observed upon siRNA-mediated knock-down of LRP/LR**.**

| **Fold change** | **Protein** | **Name** | **P- value** | **Function** |
| --- | --- | --- | --- | --- |
| 2.01 | sp\|P48735\|IDHP_HUMAN | Isocitrate dehydrogenase NADP | 0.024 | Catalyses the oxidative decarboxylation of isocitrate to 2-oxoglutarate [42] |
| 2.01 | sp\|Q9Y3D6\|FIS1_HUMAN | Fission 1 protein | 0.011 | Regulation of mitochondrial morphology, the cell cycle, autophagy and apoptosis [43] |
| 2.078 | sp\|Q53GQ0\|DHB12_HUMAN | Estradiol 17-beta-dehydrogenase 12 | 0.005 | Converts the potent oestrogen oestradiol into its less active metabolite estrone [44] |
| 2.1 | sp\|Q12981\|SEC20_HUMAN | Vesicle transport protein | 0.035 | Targets Golgi-derived retrograde transport vesicles with the ER/ Participates in intrinsic apoptotic pathway [45] |
| 2.12 | sp\|Q5JTW2\|CEP78_HUMAN | Centrosomal Protein 78 | 0.039 | Regulates of centrosome-related events during the cell cycle, and required for ciliogenesis/Tumour suppressor properties [46] |
| 2.16 | sp\|Q92597\|NDRG1_HUMAN | N-myc Downstream-Regulated Gene 1 | 0.006 | Metastasis suppressor [47] |
| 2.18 | sp\|Q9P035\|HACD3_HUMAN | 3-Hydroxyacyl-CoA Dehydratase 3 | 0.016 | Enzyme binding and lyase activity [48] |
| 2.26 | sp\|P55795\|HNRH2_HUMAN | Heterogeneous Nuclear Ribonucleoprotein H2 | 0.046 | Pre-mRNA processing, mRNA metabolism and transport [49] |
| 2.29 | sp\|Q14534\|ERG1_HUMAN | Early growth response protein 1 | 0.008 | Involved in transcription/ tumour suppressor [50] |
| 2.3 | sp\|Q7Z4H8\|PLGT3_HUMAN | Protein O-Glucosyltransferase 3 | 0.007 | Regulates Notch signalling pathway [51] |
| 2.35 | sp\|Q14728\|MFS10_HUMAN | Major Facilitator Superfamily Domain Containing 10 | 0.008 | Transmembrane transporter activity and tetracycline transmembrane transporter activity [52] |
| 2.36 | sp\|A1L0T0\|ILVBL_HUMAN | Acetolactate synthase-like protein | 0.003 | Catalytic enzyme involved in the biosynthesis of various amino acids [53] |
| 2.44 | sp\|Q7Z3K3\|POGZ_HUMAN | Pogo Transposable Element Derived with ZNF Domain/ Zinc Finger Protein 280E | 0.046 | Plays a role in mitotic cell cycle progression / nucleic acid binding [54] |
| 2.46 | sp\|Q6NW34\|NEPRO_HUMAN | Nucleolus and neural progenitor protein | 0.02 | Plays a role in Notch signalling pathway [55] |
| 2.51 | sp\|Q86SK9\|SCD5_HUMAN | Stearoyl-CoA Desaturase 5. Stearoyl-CoA desaturase | 0.025 | Catalyses the formation of monounsaturated fatty acids from saturated fatty acids [56] |
| 2.54 | sp\|O00291\|HIP1_HUMAN | Huntingtin interacting protein 1 | 0.021 | Involved in Huntingtin's disease/ proapoptotic protein (intrinsic apoptosis pathway) [57] |
| 2.55 | sp\|Q8IUX4\|ABC3F_HUMAN | Apolipoprotein B MRNA Editing Enzyme Catalytic Subunit 3F | 0.044 | RNA processing [58] |
| 2.55 | sp\|Q643R3\|LPCT4_HUMAN | Lysophosphatidylcholine Acyltransferase 4 | 0.014 | Precursor in the biosynthesis of all glycerolipids [59] |
| 2.56 | sp\|Q9ULC4\|MCTS1_HUMAN | Multiple Copies In T-Cell Lymphoma-1 | 0.026 | RNA binding and translation initiation factor activity [60] |
| 2.57 | sp\|P07305\|H10_HUMAN | H1 Histone Family Member 0 | 0.006 | Plays a role in apoptotic DNA fragmentation and chromatin silencing [61] |
| 2.61 | sp\|Q5JU69\|TOR2A_HUMAN | Torsin Family 2 Member A | 0.011 | Play roles in hypotension, myocardial growth and the induction of mitogenesis [62] |
| 2.65 | sp\|O95994\|AGR2_HUMAN | Anterior gradient protein 2 homolog | 0.006 | Proto-oncogene that may play a role in cell migration, cell differentiation and cell growth [63] |
| 2.68 | sp\|P62854\|RS26_HUMAN | Ribosomal Protein S26 | 0.019 | Regulates the MDM2/MDMX–p53 cascade, consequently suppressing tumour cell proliferation [64] |
| 2.92 | sp\|Q13228\|SBP1_HUMAN | Selenium-binding protein 1 | 0.001 | Tumour suppressor properties [65] |
| 2.94 | sp\|P06733\|ENOA_HUMAN | Enolase 1 | 0.011 | May be a tumour suppressor however, ENOA's role in tumours is controversial [66] |
| 3.09 | sp\|Q5U5X0\|LYRM7_HUMAN | LYR Motif Containing 7 | 0.035 | Main enzyme complex in the mitochondrial respiratory chain [67] |
| 3.11 | sp\|P42858\|HD_HUMAN | Huntingtin | 0.026 | Although the exact function of this protein is unknown, it may play a role in microtubule-mediated transport or vesicle function [68] |
| 3.2 | sp\|P27144\|KAD4_HUMAN | Adenylate Kinase 4 | 0.001 | GTP binding and adenylate kinase activity/Biomarker for lung metastasis [69] |
| 3.21 | sp\|P0DMM9\|ST1A3_HUMAN | Sulfotransferase Family 1A Member 3 | 0.032 | Catalyses the sulphate conjugation of many hormones, neurotransmitters, drugs [70] |
| 3.22 | sp\|Q53EL6\|PDCD4_HUMAN | Programmed cell death protein 4 | 0.001 | Inhibits translation initiation and protein synthesis (tumour suppressor properties) [71] |
| 3.32 | sp\|Q8TEU7\|RPGF6_HUMAN | Rap Guanine Nucleotide Exchange Factor 6 | 0.048 | Involved in Ras signalling and Tight junction pathways/involved in the activation of small GTPases [72] |
| 3.45 | sp\|Q9UN37\|VPS4A_HUMAN | Vacuolar protein sorting-associated protein 4A | 0.011 | Involved in intracellular protein trafficking/tumour suppressor properties [73] |
| 3.46 | sp\|Q9P0S9\|TM14C_HUMAN | Transmembrane Protein 14C | 0.024 | Pro-oncogene [74] |
| 3.51 | sp\|O00204\|ST2B1_HUMAN | Sulfotransferase family cytosolic 2B member 1 | 0.033 | Catalyses the sulphate conjugation of numerous hormones, neurotransmitters, drugs and xenobiotic compounds[75] |
| 3.68 | sp\|P22392\|NDKB_HUMAN | Nucleoside diphosphate kinase B | 0.021 | Metastasis inhibition [76] |
| 3.76 | sp\|Q9BYD2\|RM09_HUMAN | Mitochondrial Ribosomal Protein L9 | 0.013 | Structural constituent of ribosome [77] |
| 3.8 | sp\|Q9Y224\|RTRAF_HUMAN | RNA transcription, translation and transport factor protein | 0.002 | RNA transcription, translation and transport [78] |
| 3.91 | sp\|Q99707\|METH_HUMAN | Methionine synthase | 0.004 | Key enzyme involved in folate metabolism [79] |
| 3.94 | sp\|Q68EM7\|RHG17_HUMAN | Rho GTPase Activating Protein 17 | 0.009 | Rho GTPase-activating protein involved in the maintenance of tight junctions [80] |
| 4.22 | sp\|Q02818\|NUCB1_HUMAN | Nucleobindin-1 | 0.018 | Calcium homeostasis [81] |
| 4.78 | sp\|P04632\|CPNS1_HUMAN | Calpain Small Subunit 1 | 0.038 | calcium-dependent cysteine proteinase whose proteolytic activities influence apoptosis, proliferation, migration, adhesion, and autophagy [82] |
| 5.14 | sp\|Q16795\|NDUA9_HUMAN | NADH dehydrogenase (Ubiquinone) 1 alpha subcomplex subunit | 0.012 | Involved in the mitochondrial membrane respiratory chain [83] |
| 5.29 | sp\|O00442\|RTCA_HUMAN | RNA 3'-terminal phosphate cyclase | 0.003 | RNA processing [84] |
| 5.37 | sp\|P16104\|H2AX_HUMAN | Histone H2AX | 0.023 | Key role in the double-stranded break response, DNA damage leading to apoptosis [85] |

**Table S6: List of proteins involved in ribosomal processing and translation**

|  | **Fold change** | **Protein** | **Name** |
| --- | --- | --- | --- |
| **Down-regulated** |  |  |  |
|  | -4.28 | sp\|P41567\|EIF1_HUMAN | Eukaryotic translation initiation factor 1 |
|  | -3.5 | sp\|Q7Z478\|DHX29_HUMAN | DExH-box helicase 29 |
|  | -3.39 | sp\|P20042\|IF2B_HUMAN | Eukaryotic translation initiation factor 2 subunit 2 |
|  | -2.91 | sp\|P62875\|RPAB5_HUMAN | DNA-directed RNA polymerases I, II, and III subunit |
|  | -2.85 | sp\|P54105\|ICLN_HUMAN | Methylosome subunit pICln |
|  | -2.36 | sp\|P63220\|RS21_HUMAN | Ribosomal Protein S21 |
| **Up-regulated** | 2.26 | sp\|P55795\|HNRH2_HUMAN | Heterogeneous Nuclear Ribonucleoprotein H2 |
|  | 2.55 | sp\|Q8IUX4\|ABC3F_HUMAN | Apolipoprotein B MRNA Editing Enzyme Catalytic Subunit 3F |
|  | 2.56 | sp\|Q9ULC4\|MCTS1_HUMAN | Multiple Copies In T-Cell Lymphoma-1 |
|  | 3.8 | sp\|Q9Y224\|RTRAF_HUMAN | RNA transcription, translation and transport factor protein |
|  | 5.29 | sp\|O00442\|RTCA_HUMAN | RNA 3'-terminal phosphate cyclase |
|  | 3.76 | sp\|Q9BYD2\|RM09_HUMAN | Mitochondrial Ribosomal Protein L9 |

**Table S7: List of proteins involved in nuclear and chromatin maintenance**

|  | **Fold change** | **Protein** | **Name** |
| --- | --- | --- | --- |
| **Down-regulated** | -8.2 | sp\|P09651\|HNRNPA1_HUMAN | Heterogeneous nuclear ribonucleoprotein A1 |
|  | -3.32 | sp\|Q9UKI8\|TLK1_HUMAN | Serine/threonine-protein kinase tousled-like 1 |
|  | -5.62 | sp\|O96028\|NSD2_HUMAN | Histone-lysine N-methyltransferase |
|  | -4.95 | sp\|Q9UKY7\|CDV3_HUMAN | Protein CDV3 homolog |
|  | -2.52 | sp\|Q15370\|ELOB_HUMAN | Elongin B |
|  | -2.44 | sp\|O15405\|TOX3_HUMAN | TOX high mobility group box family member 3 |

**Table S8: List of proteins involved in cytoskeletal maintenance and cell anchorage**

|  | **Fold change** | **Protein** | **Name** |
| --- | --- | --- | --- |
|  | -5.75 | sp\|Q14244\|MAP7_HUMAN | Ensconsin |
| **Down-regulated** | -4.39 | sp\|Q9UK76\|JUPI1_HUMAN | Jupiter Microtubule Associated Homolog 1 |
|  | -4.26 | sp\|O14530\|TXND9_HUMAN | Thioredoxin Domain Containing 9 |
|  | -4.01 | sp\|Q14118\|DAG1_HUMAN | Dystroglycan |
|  | -3.12 | sp\|P15151\|PVR_HUMAN | Poliovirus receptor/ PVR Cell Adhesion Molecule |
|  | -3.32 | sp\|Q6QEF8\|CORO6_HUMAN | Coronin-like protein E |
|  | -2.95 | sp\|P02751\|FINC_HUMAN | Fibronectin 1 |
|  | -2.92 | sp\|Q9Y639\|NPTN_HUMAN | Neuroplastin |
|  | -2.79 | sp\|Q06481\|APLP2_HUMAN | Amyloid-like protein 2 |
|  | -2.53 | sp\|Q99439\|CNN2_HUMAN | Calponin-2 |
|  | -2.17 | sp\|Q15417\|CNN3_HUMAN | Calponin-3 |
|  | -2.37 | sp\|Q86UK7\|ZN598_HUMAN | Zinc Finger Protein 598 |
| **Up-regulated** | 2.01 | sp\|Q9Y3D6\|FIS1_HUMAN | Fission 1 protein |
|  | 2.3 | sp\|Q7Z4H8\|PLGT3_HUMAN | Protein O-Glucosyltransferase 3 |
|  | 2.44 | sp\|Q7Z3K3\|POGZ_HUMAN | Pogo Transposable Element Derived with ZNF Domain/ Zinc Finger Protein 280E |
|  | 2.46 | sp\|Q6NW34\|NEPRO_HUMAN | Nucleolus and neural progenitor protein |
|  | 2.65 | sp\|O95994\|AGR2_HUMAN | Anterior gradient protein 2 homolog |
|  | 3.46 | sp\|Q9P0S9\|TM14C_HUMAN | Transmembrane Protein 14C |

**Table S9: List of proteins involved in vesicle transport and membrane trafficking**

| **Down-regulated** | **Fold change** | **Protein** | **Name** |
| --- | --- | --- | --- |
|  | -4.71 | sp\|Q9HD42\|CHM1A_HUMAN | Charged Multivesicular Body |
|  | -3.94 | sp\|Q9NWW5\|CLN6_HUMAN | Ceroid-lipofuscinosis neuronal protein 6 |
|  | -2.62 | sp\|Q8TAT6\|NPL4_HUMAN | Nuclear protein localization protein 4 |
| **Up-regulated** | -2.06 | sp\|Q5JRA6\|TGO1_HUMAN | Transport and Golgi organization protein 1/Melanoma Inhibitory Activity Protein 3 |
|  | 2.1 | sp\|Q12981\|SEC20_HUMAN | Vesicle transport protein |
|  | 2.35 | sp\|Q14728\|MFS10_HUMAN | Major Facilitator Superfamily Domain Containing 10 |
|  | 3.11 | sp\|P42858\|HD_HUMAN | Huntingtin |

**Table S10: List of proteins involved in apoptotic regulation**

| **Down-regulated** | **Fold change** | **Protein** | **Name** |
| --- | --- | --- | --- |
|  | -4.63 | sp\|Q9NYF8\|BCLF1_HUMAN | Bcl-2-associated transcription factor 1 |
|  | -4.43 | sp\|Q7L9L4\|MOB1B_HUMAN | Mps one binder kinase activator-like 1A |
|  | -4.02 | sp\|P41222\|PTGDS_HUMAN | Prostaglandin-H2 D-isomerase |
|  | -2.79 | sp\|O60610\|DIAP1_HUMAN | Death-associated inhibitor of apoptosis 1 |
|  | -2.4 | sp\|Q13546\|RIPK1_HUMAN | Receptor-interacting serine/threonine-protein kinase 1 |
| **Up-regulated** | -2.12 | sp\|P07203\|GPX1_HUMAN | Glutathione peroxidase 1 |
|  | 2.12 | sp\|Q5JTW2\|CEP78_HUMAN | Centrosomal Protein 78 |
|  | 2.16 | sp\|Q92597\|NDRG1_HUMAN | N-myc Downstream-Regulated Gene 1 |
|  | 2.29 | sp\|Q14534\|ERG1_HUMAN | Early growth response protein 1 |
|  | 2.54 | sp\|O00291\|HIP1_HUMAN | Huntingtin interacting protein 1 |
|  | 2.57 | sp\|P07305\|H10_HUMAN | H1 Histone Family Member 0 |
|  | 2.68 | sp\|P62854\|RS26_HUMAN | Ribosomal Protein S26 |
|  | 2.92 | sp\|Q13228\|SBP1_HUMAN | Selenium-binding protein 1 |
|  | 2.94 | sp\|P06733\|ENOA_HUMAN | Enolase 1 |
|  | 3.22 | sp\|Q53EL6\|PDCD4_HUMAN | Programmed cell death protein 4 |
|  | 3.45 | sp\|Q9UN37\|VPS4A_HUMAN | Vacuolar protein sorting-associated protein 4A |
|  | 3.68 | sp\|P22392\|NDKB_HUMAN | Nucleoside diphosphate kinase B |
|  | 5.14 | sp\|Q16795\|NDUA9_HUMAN | NADH dehydrogenase (Ubiquinone) 1 alpha subcomplex subunit |
|  | 5.37 | sp\|P16104\|H2AX_HUMAN | Histone H2AX |

**Table S11: List of proteins involved in autophagic regulation**

| **Up-regulated** | **Fold change** | **Protein** | **Name** |
| --- | --- | --- | --- |
|  | 2.01 | sp\|P48735\|IDHP_HUMAN | Isocitrate dehydrogenase NADP |
|  | 2.078 | sp\|Q53GQ0\|DHB12_HUMAN | Estradiol 17-beta-dehydrogenase 12 |
|  | 2.18 | sp\|Q9P035\|HACD3_HUMAN | 3-Hydroxyacyl-CoA Dehydratase 3 |
|  | 2.36 | sp\|A1L0T0\|ILVBL_HUMAN | Acetolactate synthase-like protein |
|  | 2.51 | sp\|Q86SK9\|SCD5_HUMAN | Stearoyl-CoA Desaturase 5. Stearoyl-CoA desaturase |
|  | 2.55 | sp\|Q643R3\|LPCT4_HUMAN | Lysophosphatidylcholine Acyltransferase 4 |
|  | 3.09 | sp\|Q5U5X0\|LYRM7_HUMAN | LYR Motif Containing 7 |
|  | 3.2 | sp\|P27144\|KAD4_HUMAN | Adenylate Kinase 4 |
|  | 3.21 | sp\|P0DMM9\|ST1A3_HUMAN | Sulfotransferase Family 1A Member 3 |
|  | 3.51 | sp\|O00204\|ST2B1_HUMAN | Sulfotransferase family cytosolic 2B member 1 |
|  | 4.22 | sp\|Q02818\|NUCB1_HUMAN | Nucleobindin-1 |
|  | 4.78 | sp\|P04632\|CPNS1_HUMAN | Calpain Small Subunit 1 |

**Table S12: List of remaining proteins identified (no direct link to LRP/LR)**

|  | **Fold change** | **Protein** | **Name** |
| --- | --- | --- | --- |
| **Down-regulated** | -2.5 | sp\|P61916\|NPC2_HUMAN | Intracellular cholesterol transporter 2 |
|  | -4.48 | sp\|Q9H832\|UBE2Z_HUMAN | Ubiquitin conjugating enzyme E2 Z |
|  | -3.29 | sp\|Q8N806\|UBR7_HUMAN | Putative E3 ubiquitin-protein ligase |
|  | -2.43 | sp\|O96005\|CLPT1_HUMAN | ATP-dependent Clp protease ATP-binding subunit |
|  | -4.64 | sp\|P17152\|TMM11_HUMAN | Transmembrane protein 11 |
|  | -3.45 | sp\|Q9UJC5\|SH3L2_HUMAN | SH3 domain-binding glutamic acid-rich-like protein |
| **Up-regulated** | 3.91 | sp\|Q99707\|METH_HUMAN | Methionine synthase |
|  | 2.61 | sp\|Q5JU69\|TOR2A_HUMAN | Torsin Family 2 Member A |
|  | 3.32 | sp\|Q8TEU7\|RPGF6_HUMAN | Rap Guanine Nucleotide Exchange Factor 6 |
|  | 3.94 | sp\|Q68EM7\|RHG17_HUMAN | Rho GTPase Activating Protein 17 |


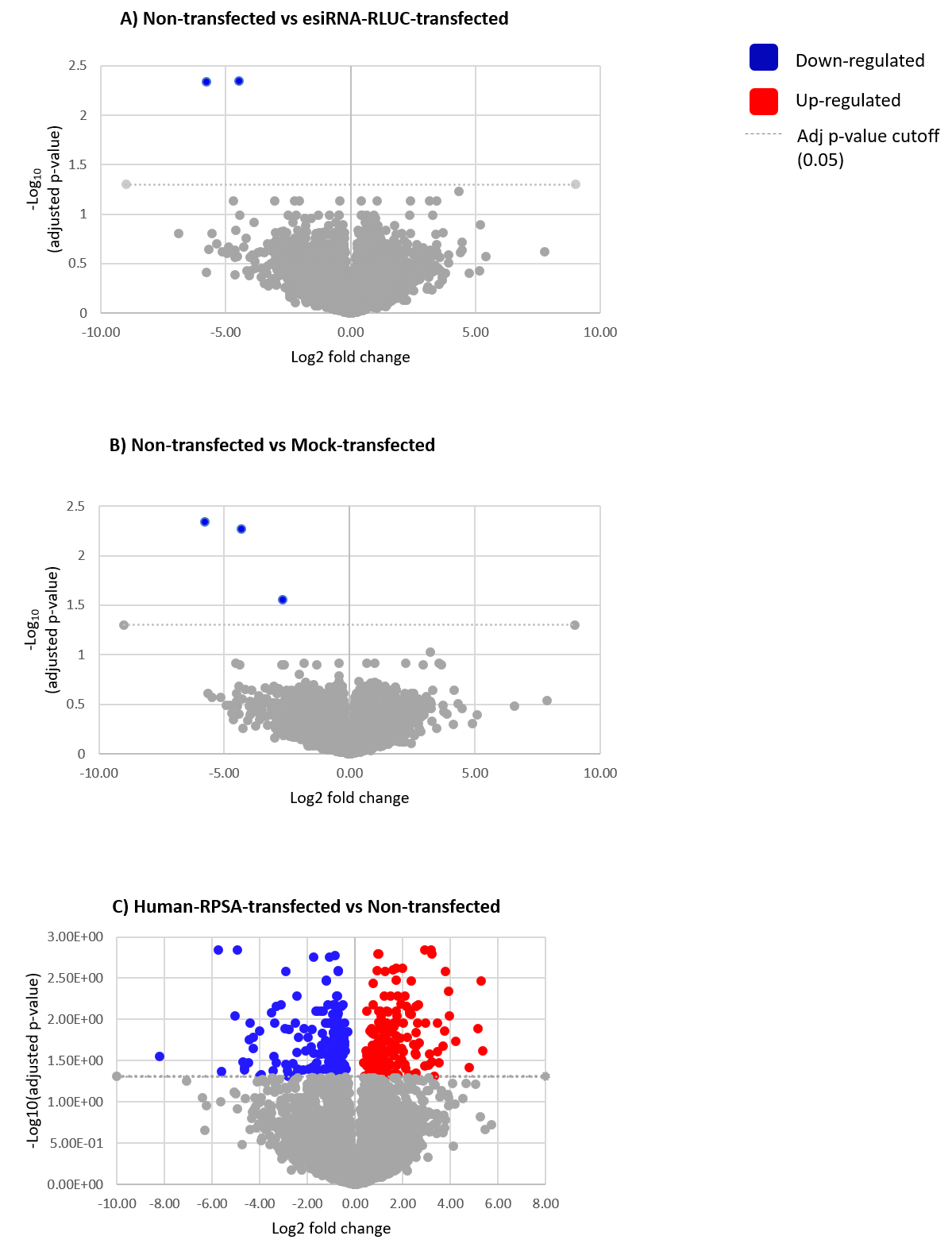


Figure S15: Proteins found to be affected upon siRNA-mediated knock-down of LRP/LR using SWATH-MS based proteomics in late (DLD-1) stage colorectal cancer cells**. A) Non-transfected vs esiRNA-RLUC samples.** When the non-transfected and negative control esiRNA-RLUC samples were compared to one another, only two proteins were significantly affected. **Non-transfected vs mock-transfected samples.** When the non-transfected cells were compared to the mock-transfected control samples, only three proteins were found to be significantly affected, confirming that the transfection reagent does not have an effect. **C)** **Human-RPSA siRNA-transfected vs non-transfected samples.** It can be seen that when Human-RPSA siRNA transfected samples were compared to non-transfected samples, it showed a large change in the DLD-1 cells proteome with several proteins being either down- or and up-regulated, displaying a similar effect to when the Human-RPSA siRNA-transfected samples were compared to the negative control esiRNA-RLUC-transfected samples.

**Primers used for mRNA quantification in qPCR reactions:**

**Table S11: List of primers used for mRNA quantification through qPCR**

| **Gene** | **Forward Primer (5’-3’)** | **Reverse Primer (3’-5’)** |
| --- | --- | --- |
| GAPDH (Inqaba Biotech™) (Deng et al, 2014 [86] | GTGGACCTGACCTGCCGTCT | GGAGGAGTGGGTGTCGCTGT |
| ACTB (Integrated DNA Technologies®) - original synthesis | AGTTGCGTTACACCCTTTC | CCTTCACCGTTCCAGTTT |
| LRP/LR (Integrated DNA Technologies®) - original synthesis | GCCATTGAAAACCCTGCTGA | AGCGCAATGGTAGGTAGGTT |
| p53 (Inqaba Biotech™) (Chew et al, 2012 [87]) | TAACAGTTCCTGCATGGGCGGC | AGGACAGGCACAAACACGCACC |
| Bax (Integrated DNA Technologies®) - original synthesis) | AAGAAGCTGAGCGAGTGTCT | GTTCTGATCAGTTCCGGCAC |
| Bcl-2 (Inqaba Biotech™)  (Jackson et al, 2006 [88]) | CCTGATTCATTGGGAAGTTTCAA | AAACAAATGCATAAGGCAACGA |
| TERT (Integrated DNA Technologies®) – original synthesis | CTTCCTACGCTTCATGTGCC | AATCATCCACCAAACGCAGG |
| CREB (Inqaba Biotech™) (Shankar et al, 2005 [89]) | AAGCTGAAAGTCAACAAATGACAGTT | TGGACTGTCTGCCCATTGG |

## Reagents and Materials

- Media (DMEM)- GE Healthcare Life sciences (Massachusetts, USA)
- BSA, Ethanol -VWR Life sciences (Pennsylvania, USA)
- Molecular weight marker, OneTaq® HotStart Taq Polymerase - New England Biolabs Inc. (Massachusetts, USA)
- Penicillin/streptomycin, PBS, Opti-MEM – Gibco Life Sciences (California, USA)
- Tween-20, DMSO, lysis buffer - Merck Millipore (Johannesburg, RSA)
- Ethanol, Methanol, Paraformaldehyde, Acetic acid -Associate chemical enterprise (ACE) (Johannesburg, RSA)
- MTT powder - Duchefa Biochemie (Haarlem, The Netherlands)
- Trypsin/EDTA, Pen/Strep Amphoteracin B 100X – Lonza (Basel, Switzerland)
- HEPES – Thermo Fisher Scientific (Massachusetts, USA)
- Hoechst-33258 - Invitrogen (Oregon, USA)
- iRT peptide standards – Biognosys (Schlieran, Switzerland)
- Agarose Csl-AG100 LE - Cleaver Scientific Ltd, (Warwickshire, UK)
- Non-essential amino acids, BCA reagents, APS, acrylamide, TEMED, fluoromount mounting fluid, SDS, Triton-X, CHAPS lysis buffer, Agarose gel stain, Glycine, Loading buffer, 2ml centrifugation tubes, β-mercaptoethanol, Bisacrylamide, Trypan Blue, PCA , FCS – Sigma Life science (Missouri, USA)
- PVDF membrane – Pall corporation (New York, USA)
- Chemiluminescent substrate, Cell counting slides – Biorad (California, USA)
- Microscopy slides and coverslips – Labocare (Gauteng, RSA)
- Matrigel Matrix, 24-well, 6-well plates, 15ml and 50 ml falcon tubes – Corning Inc. (New York, USA)
- 25 and 75 cm^3^ cell culture flasks - NEST Biotechnology Co. Lt (Wuxi, China)
- 0.2 ml PCR tubes – STARLAB (Hamburg, Germany)
- Guava® cell cycle reagent - Luminex Corporation (Texas, USA)

Antibodies and siRNAs

- IgG1-iS18 (1:1000) – Affimed Therapeutics (Heidelberg, Germany)
- Anti-human IgG-HRP, anti-human PE, anti-rabbit APC– Abcam (Cambridge, UK)
- Anti-rabbit pTERT (1:1000), Anti-β actin peroxidise (1:1000), esiRNA-RPSA (200ng/ul), esiRNA-RLUC (200 ng/ul) – Sigma Life Sciences (Missouri, USA)
- Human-RPSA siRNA(5 nmol/20nmol) and DharmaFect1 transfection reagent – GE Life Sciences (Massachusetts, USA)
- Anti-rabbit TRF-1 (1:1000), Anti-rabbit IgG-HRP (1:2000), Anti-mouse IgG-HRP (1:2000) – Cell Signalling Technology® (Massachusetts, USA)
- Anti-rabbit TRF-2 (1:1000) – Merck Millipore (Gauteng, RSA)
- Anti-mouse hTERT (1:1000) – Invitrogen (California, USA)

## Kits

- Annexin V-FITC/ PI kit – BD Biosciences (New Jersey, USA)
- Caspase 3,-8 and -9 kits and cell cycle kit – Merck Millipore (Gauteng, RSA)
- MAPK, Phospho-MAPK and Apoptosis Proteome Profiler Antibody Array™ kits – R&D Systems (Minnesota, USA)
- SensiFAST SYBR™ No-ROX kit, SensiFAST™ cDNA synthesis kit – Bioline (London, UK)
- KAPA Taq PCR kit – Kapa Biosystems (Massachusetts, USA)
- Quick-RNA™ MiniPrep Plus kit – Zymo Research (California, USA)

## Equipment list

- Flow cytometer BD Accuri C - BD Biosciences (California, USA)
- Confocal microscope – Zeiss LSM 710 3-channel (images were captured using the blue laser, 63X magnification, and using Zen 2011 software). (Oberkochen, Germany)
- Nanodrop® ND-1000 - NanoDrop Technologies (Detroit, USA)
- KingFisher™ Flex magnetic particle processing robot, Dionex Ultimate 3000 RSLC system coupled to an AB Sciex 6600 TipleTOF mass spectrometer, Bright field microscope/EVOS Floid cell imaging station (Massachusetts, USA)
- Laminar flow – Labotec (Gauteng, RSA)
- ELISA reader – Tecan (using Magellan software) (Shanghai, China)
- Eppendorf 5417C - Merck Millipore (Gauteng, RSA)
- Pipettes and micropipettes – Eppendorf research (Hamburg, Germany)
- TC20 cell counter, gel casting and running apparatus, Chemidoc imaging machine, Trans-Blot® Turbo™ Transfer System, CFX Maestro™ thermo cycler – Biorad (California, USA)
- pH meter – Eutech instruments (Singapore, Republic of Singapore)

Software

- ImageJ (version 1.8)
- BD Sciences flow cytometry software - BD Biosciences (California. USA)
- CFX Maestro™ (version 1.0), Image Lab (version 5.1) Image acquisition and analysis software -BioRad (California, USA)
- BindIt Software 3.0 - Thermo Fisher scientific (Massachusetts, USA)
- Protein Pilot (version 5.0.1)
- Skyline (version 4.1.1.18179) spectral library builder
- Proteo Wizard MS Convert
- GraphPad prism (version 5.03)
- Microsoft® Excel 365

**References**

1. Vania L, Rebelo TM, Ferreira E, Weiss SFT: **Knock-down of LRP/LR promotes apoptosis in early and late stage colorectal carcinoma cells via caspase activation**. *BMC Cancer* 2018, **18**(1):602.

2. Sundquist T, Moravec R, Niles A, O’Brien M, Riss T: **Timing your apoptosis assays**. *Cell Notes* 2006, **16**:18-21.

3. Paronetto MP, Achsel T, Massiello A, Chalfant CE, Sette C: **The RNA-binding protein Sam68 modulates the alternative splicing of Bcl-x**. *J Cell Biol* 2007, **176**(7):929-939.

4. Treiber T, Treiber N, Plessmann U, Harlander S, Daiß J-L, Eichner N, Lehmann G, Schall K, Urlaub H, Meister G: **A compendium of RNA-binding proteins that regulate microRNA biogenesis**. *Molecular cell* 2017, **66**(2):270-284. e213.

5. Bulinski JC, Odde DJ, Howell BJ, Salmon TD, Waterman-Storer CM: **Rapid dynamics of the microtubule binding of ensconsin in vivo**. *Journal of cell science* 2001, **114**(21):3885-3897.

6. Bhat KM, Setaluri V: **Microtubule-associated proteins as targets in cancer chemotherapy**. *Clinical Cancer Research* 2007, **13**(10):2849-2854.

7. Garlisi CG, Uss AS, Xiao H, Tian F, Sheridan KE, Wang L, Motasim Billah M, Egan RW, Stranick KS, Umland SP: **A unique mRNA initiated within a middle intron of WHSC1/MMSET encodes a DNA binding protein that suppresses human IL-5 transcription**. *American Journal of Respiratory Cell and Molecular Biology* 2001, **24**(1):90-98.

8. de Hostos EL: **The coronin family of actin-associated proteins**. *Trends in cell biology* 1999, **9**(9):345-350.

9. Xiao H, Zhou B, Jiang N, Cai Y, Liu X, Shi Z, Li M, Du C: **The potential value of CDV3 in the prognosis evaluation in Hepatocellular carcinoma**. *Genes & diseases* 2018, **5**(2):167-171.

10. Howard TL, Stauffer DR, Degnin CR, Hollenberg SMJJocs: **CHMP1 functions as a member of a newly defined family of vesicle trafficking proteins**. 2001, **114**(13):2395-2404.

11. Rival T, Macchi M, Arnauné‐Pelloquin L, Poidevin M, Maillet F, Richard F, Fatmi A, Belenguer P, Royet JJEr: **Inner‐membrane proteins PMI/TMEM11 regulate mitochondrial morphogenesis independently of the DRP1/MFN fission/fusion pathways**. 2011, **12**(3):223-230.

12. Zhou X, Li X, Cheng Y, Wu W, Xie Z, Xi Q, Han J, Wu G, Fang J, Feng YJNc: **BCLAF1 and its splicing regulator SRSF10 regulate the tumorigenic potential of colon cancer cells**. 2014, **5**:4581.

13. Baarends WM, Roest HP, Grootegoed JAJM, endocrinology c: **The ubiquitin system in gametogenesis**. 1999, **151**(1-2):5-16.

14. Xiong S, Couzens AL, Kean MJ, Mao DY, Guettler S, Kurinov I, Gingras A-C, Sicheri FJM, Proteomics C: **Regulation of protein interactions by Mps One Binder (MOB1) phosphorylation**. 2017, **16**(6):1111-1125.

15. Ali MU, Ur Rahman MS, Jia Z, Jiang C: **Eukaryotic translation initiation factors and cancer**. *Tumor Biology* 2017, **39**(6):1010428317709805.

16. Chen D, Zou J, Zhao Z, Tang X, Deng Z, Jia J, Liu SJCd, disease: **TXNDC9 promotes hepatocellular carcinoma progression by positive regulation of MYC-mediated transcriptional network**. 2018, **9**(11):1110.

17. Shyu R-Y, Wu C-C, Wang C-H, Tsai T-C, Wang L-K, Chen M-L, Jiang S-Y, Tsai F-MJJobs: **H-rev107 regulates prostaglandin D2 synthase-mediated suppression of cellular invasion in testicular cancer cells**. 2013, **20**(1):30.

18. Sgambato A, Migaldi M, Montanari M, Camerini A, Brancaccio A, Rossi G, Cangiano R, Losasso C, Capelli G, Trentini GP: **Dystroglycan expression is frequently reduced in human breast and colon cancers and is associated with tumor progression**. *The American journal of pathology* 2003, **162**(3):849-860.

19. Heine C, Koch B, Storch S, Kohlschütter A, Palmer DN, Braulke TJJoBC: **Defective endoplasmic reticulum-resident membrane protein CLN6 affects lysosomal degradation of endocytosed arylsulfatase A**. 2004, **279**(21):22347-22352.

20. Parsyan A, Shahbazian D, Martineau Y, Petroulakis E, Alain T, Larsson O, Mathonnet G, Tettweiler G, Hellen CU, Pestova TVJPOTNAOS: **The helicase protein DHX29 promotes translation initiation, cell proliferation, and tumorigenesis**. 2009, **106**(52):22217-22222.

21. Mazzocco M, Maffei M, Egeo A, Vergano A, Arrigo P, Di Lisi R, Ghiotto F, Scartezzini PJG: **The identification of a novel human homologue of the SH3 binding glutamic acid-rich (SH3BGR) gene establishes a new family of highly conserved small proteins related to Thioredoxin Superfamily**. 2002, **291**(1-2):233-239.

22. Lu Y, Cai Z, Galson DL, Xiao G, Liu Y, George DE, Melhem MF, Yao Z, Zhang JJTP: **Monocyte chemotactic protein‐1 (MCP‐1) acts as a paracrine and autocrine factor for prostate cancer growth and invasion**. 2006, **66**(12):1311-1318.

23. Lee S-B, Segura-Bayona S, Villamor-Payà M, Saredi G, Todd MA, Attolini CS-O, Chang T-Y, Stracker TH, Groth A: **Tousled-like kinases stabilize replication forks and show synthetic lethality with checkpoint and PARP inhibitors**. *Science advances* 2018, **4**(8):eaat4985.

24. Tasaki T, Mulder LC, Iwamatsu A, Lee MJ, Davydov IV, Varshavsky A, Muesing M, Kwon YTJM, biology c: **A family of mammalian E3 ubiquitin ligases that contain the UBR box motif and recognize N-degrons**. 2005, **25**(16):7120-7136.

25. Bowers JR, Readler JM, Sharma P, Excoffon KJJVr: **Poliovirus receptor: more than a simple viral receptor**. 2017, **242**:1-6.

26. Mouw JK, Ou G, Weaver VMJNrMcb: **Extracellular matrix assembly: a multiscale deconstruction**. 2014, **15**(12):771.

27. Beesley PW, Herrera‐Molina R, Smalla KH, Seidenbecher CJJon: **The Neuroplastin adhesion molecules: key regulators of neuronal plasticity and synaptic function**. 2014, **131**(3):268-283.

28. Kershnar E, Wu S-Y, Chiang C-MJJoBC: **Immunoaffinity purification and functional characterization of human transcription factor IIH and RNA polymerase II from clonal cell lines that conditionally express epitope-tagged subunits of the multiprotein complexes**. 1998, **273**(51):34444-34453.

29. Chari A, Golas MM, Klingenhäger M, Neuenkirchen N, Sander B, Englbrecht C, Sickmann A, Stark H, Fischer UJC: **An assembly chaperone collaborates with the SMN complex to generate spliceosomal SnRNPs**. 2008, **135**(3):497-509.

30. Pandey P, Rachagani S, Das S, Seshacharyulu P, Sheinin Y, Naslavsky N, Pan Z, Smith BL, Peters HL, Radhakrishnan PJO: **Amyloid precursor-like protein 2 (APLP2) affects the actin cytoskeleton and increases pancreatic cancer growth and metastasis**. 2015, **6**(4):2064.

31. Khan F, Fujioka M, Datta P, Fernandes-Alnemri T, Jaynes J, Alnemri EJCd, differentiation: **The interaction of DIAP1 with dOmi/HtrA2 regulates cell death in Drosophila**. 2008, **15**(6):1073.

32. Bays NW, Wilhovsky SK, Goradia A, Hodgkiss-Harlow K, Hampton RYJMbotc: **HRD4/NPL4 is required for the proteasomal processing of ubiquitinated ER proteins**. 2001, **12**(12):4114-4128.

33. Liu R, Jin J-P: **Calponin isoforms CNN1, CNN2 and CNN3: regulators for actin cytoskeleton functions in smooth muscle and non-muscle cells**. *Gene* 2016, **585**(1):143-153.

34. Garrett KP, Aso T, Bradsher JN, Foundling SI, Lane WS, Conaway RC, Conaway JWJPotNAoS: **Positive regulation of general transcription factor SIII by a tailed ubiquitin homolog**. 1995, **92**(16):7172-7176.

35. Liao Y-J, Lin M-W, Yen C-H, Lin Y-T, Wang C-K, Huang S-F, Chen K-H, Yang C-P, Chen T-L, Hou M-FJPO: **Characterization of Niemann-Pick Type C2 protein expression in multiple cancers using a novel NPC2 monoclonal antibody**. 2013, **8**(10):e77586.

36. Han N, Li W, Zhang M: **The function of the RNA-binding protein hnRNP in cancer metastasis**. *Journal of cancer research and therapeutics* 2013, **9**(7):129.

37. Sjögren LL, Clarke AKJTPC: **Assembly of the chloroplast ATP-dependent Clp protease in Arabidopsis is regulated by the ClpT accessory proteins**. 2011, **23**(1):322-332.

38. Lin Y: **RIP1-mediated signaling pathways in cell survival and death control**. In: *Necrotic Cell Death.* edn.: Springer; 2014: 23-43.

39. Rabl J, Leibundgut M, Ataide SF, Haag A, Ban N: **Crystal structure of the eukaryotic 40S ribosomal subunit in complex with initiation factor 1**. *Science* 2011, **331**(6018):730-736.

40. Higashi Y, Pandey A, Goodwin B, Delafontaine PJBeBA-MBoD: **Insulin-like growth factor-1 regulates glutathione peroxidase expression and activity in vascular endothelial cells: Implications for atheroprotective actions of insulin-like growth factor-1**. 2013, **1832**(3):391-399.

41. Bard F, Casano L, Mallabiabarrena A, Wallace E, Saito K, Kitayama H, Guizzunti G, Hu Y, Wendler F, DasGupta RJN: **Functional genomics reveals genes involved in protein secretion and Golgi organization**. 2006, **439**(7076):604.

42. Smolková K, Ježek PJIjocb: **The role of mitochondrial NADPH-dependent isocitrate dehydrogenase in cancer cells**. 2012, **2012**.

43. Lee S, Park Y-Y, Kim S-H, Nguyen OTK, Yoo Y-S, Chan GK, Sun X, Cho HJC, sciences ml: **Human mitochondrial Fis1 links to cell cycle regulators at G2/M transition**. 2014, **71**(4):711-725.

44. Aka JA, Zerradi M, Houle F, Huot J, Lin S-XJBCR: **17beta-hydroxysteroid dehydrogenase type 1 modulates breast cancer protein profile and impacts cell migration**. 2012, **14**(3):R92.

45. Lakatos Z, Lőrincz P, Szabó Z, Benkő P, Kenéz LA, Csizmadia T, Juhász GJC: **Sec20 Is Required for Autophagic and Endocytic Degradation Independent of Golgi-ER Retrograde Transport**. 2019, **8**(8):768.

46. Zhang M, Duan T, Wang L, Tang J, Luo R, Zhang R, Kang TJCjoc: **Low expression of centrosomal protein 78 (CEP78) is associated with poor prognosis of colorectal cancer patients**. 2016, **35**(1):62.

47. Stein S, Thomas EK, Herzog B, Westfall MD, Rocheleau JV, Jackson RS, Wang M, Liang PJJoBC: **NDRG1 is necessary for p53-dependent apoptosis**. 2004, **279**(47):48930-48940.

48. Ikeda M, Kanao Y, Yamanaka M, Sakuraba H, Mizutani Y, Igarashi Y, Kihara AJFl: **Characterization of four mammalian 3‐hydroxyacyl‐CoA dehydratases involved in very long‐chain fatty acid synthesis**. 2008, **582**(16):2435-2440.

49. Castello A, Fischer B, Eichelbaum K, Horos R, Beckmann BM, Strein C, Davey NE, Humphreys DT, Preiss T, Steinmetz LMJC: **Insights into RNA biology from an atlas of mammalian mRNA-binding proteins**. 2012, **149**(6):1393-1406.

50. Hashimoto H, Olanrewaju YO, Zheng Y, Wilson GG, Zhang X, Cheng XJG, development: **Wilms tumor protein recognizes 5-carboxylcytosine within a specific DNA sequence**. 2014, **28**(20):2304-2313.

51. Takeuchi H, Schneider M, Williamson DB, Ito A, Takeuchi M, Handford PA, Haltiwanger RSJPotNAoS: **Two novel protein O-glucosyltransferases that modify sites distinct from POGLUT1 and affect Notch trafficking and signaling**. 2018, **115**(36):E8395-E8402.

52. Ushijima H, Hiasa M, Namba T, Hwang H-J, Hoshino T, Mima S, Tsuchiya T, Moriyama Y, Mizushima TJB, communications br: **Expression and function of TETRAN, a new type of membrane transporter**. 2008, **374**(2):325-330.

53. Joutel A, Ducros A, Alamowitch S, Cruaud C, Domenga V, Marechal E, Vahedi K, Chabriat H, Bousser M, Tournier-Lasserve EJG: **A human homolog of bacterial acetolactate synthase genes maps within the CADASIL critical region**. 1996, **38**(2):192-198.

54. Nozawa R-S, Nagao K, Masuda H-T, Iwasaki O, Hirota T, Nozaki N, Kimura H, Obuse CJNcb: **Human POGZ modulates dissociation of HP1α from mitotic chromosome arms through Aurora B activation**. 2010, **12**(7):719.

55. Muroyama Y, Saito TJD: **Identification of Nepro, a gene required for the maintenance of neocortex neural progenitor cells downstream of Notch**. 2009, **136**(23):3889-3893.

56. Tan S-H, Shui G, Zhou J, Shi Y, Huang J, Xia D, Wenk MR, Shen H-MJA: **Critical role of SCD1 in autophagy regulation via lipogenesis and lipid rafts-coupled AKT-FOXO1 signaling pathway**. 2014, **10**(2):226-242.

57. Hackam AS, Yassa AS, Singaraja R, Metzler M, Gutekunst C-A, Gan L, Warby S, Wellington CL, Vaillancourt J, Chen NJJoBC: **Huntingtin interacting protein 1 induces apoptosis via a novel caspase-dependent death effector domain**. 2000, **275**(52):41299-41308.

58. Gao J, Choudhry H, Cao WJCs: **Apolipoprotein B mRNA editing enzyme catalytic polypeptide‐like family genes activation and regulation during tumorigenesis**. 2018, **109**(8):2375-2382.

59. Ye G-m, Chen C, Huang S, Han D-d, Guo J-h, Wan B, Yu LJDS: **Cloning and characterization a novel human 1-acyl-sn-glycerol-3-phosphate acyltransferase gene AGPAT7**. 2005, **16**(5):386-390.

60. Levenson AS, Thurn KE, Simons LA, Veliceasa D, Jarrett J, Osipo C, Jordan VC, Volpert OV, Satcher RL, Gartenhaus RBJCr: **MCT-1 oncogene contributes to increased in vivo tumorigenicity of MCF7 cells by promotion of angiogenesis and inhibition of apoptosis**. 2005, **65**(23):10651-10656.

61. Di Liegro C, Schiera G, Di Liegro IJG: **H1. 0 Linker Histone as an Epigenetic Regulator of Cell Proliferation and Differentiation**. 2018, **9**(6):310.

62. Shichiri M, Ishimaru S, Ota T, Nishikawa T, Isogai T, Hirata YJNm: **Salusins: newly identified bioactive peptides with hemodynamic and mitogenic activities**. 2003, **9**(9):1166.

63. Kani K, Malihi PD, Jiang Y, Wang H, Wang Y, Ruderman DL, Agus DB, Mallick P, Gross MEJTP: **Anterior gradient 2 (AGR2): Blood‐based biomarker elevated in metastatic prostate cancer associated with the neuroendocrine phenotype**. 2013, **73**(3):306-315.

64. Zhou X, Liao W-J, Liao J-M, Liao P, Lu HJJomcb: **Ribosomal proteins: functions beyond the ribosome**. 2015, **7**(2):92-104.

65. Yang W, Diamond AMJBr: **Selenium-binding protein 1 as a tumor suppressor and a prognostic indicator of clinical outcome**. 2013, **1**(1):15.

66. Milone MR, Pucci B, Colangelo T, Lombardi R, Iannelli F, Colantuoni V, Sabatino L, Budillon AJMo: **Proteomic characterization of peroxisome proliferator-activated receptor-γ (PPARγ) overexpressing or silenced colorectal cancer cells unveils a novel protein network associated with an aggressive phenotype**. 2016, **10**(8):1344-1362.

67. Maio N, Kim KS, Singh A, Rouault TAJCm: **A single adaptable cochaperone-scaffold complex delivers nascent iron-sulfur clusters to mammalian respiratory chain complexes I–III**. 2017, **25**(4):945-953. e946.

68. Martin DD, Heit RJ, Yap MC, Davidson MW, Hayden MR, Berthiaume LGJHmg: **Identification of a post-translationally myristoylated autophagy-inducing domain released by caspase cleavage of huntingtin**. 2014, **23**(12):3166-3179.

69. Liu R, Ström A-L, Zhai J, Gal J, Bao S, Gong W, Zhu HJTijob, biology c: **Enzymatically inactive adenylate kinase 4 interacts with mitochondrial ADP/ATP translocase**. 2009, **41**(6):1371-1380.

70. Hildebrandt MA, Salavaggione OE, Martin YN, Flynn HC, Jalal S, Wieben ED, Weinshilboum RMJB, communications br: **Human SULT1A3 pharmacogenetics: gene duplication and functional genomic studies**. 2004, **321**(4):870-878.

71. Yang H-S, Knies JL, Stark C, Colburn NHJO: **Pdcd4 suppresses tumor phenotype in JB6 cells by inhibiting AP-1 transactivation**. 2003, **22**(24):3712.

72. Gao X, Satoh T, Liao Y, Song C, Hu C-D, Kariya K-i, Kataoka TJJoBC: **Identification and characterization of RA-GEF-2, a Rap guanine nucleotide exchange factor that serves as a downstream target of M-Ras**. 2001, **276**(45):42219-42225.

73. Takahashi Y, He H, Tang Z, Hattori T, Liu Y, Young MM, Serfass JM, Chen L, Gebru M, Chen CJNc: **An autophagy assay reveals the ESCRT-III component CHMP2A as a regulator of phagophore closure**. 2018, **9**(1):2855.

74. Schmit K, Michiels CJFip: **TMEM Proteins in Cancer: A Review**. 2018, **9**.

75. He D, Falany CNJDm, disposition: **Characterization of proline-serine-rich carboxyl terminus in human sulfotransferase 2B1b: immunogenicity, subcellular localization, kinetic properties, and phosphorylation**. 2006, **34**(10):1749-1755.

76. Liu Y-f, Yang A, Liu W, Wang C, Wang M, Zhang L, Wang D, Dong J-f, Li MJPo: **NME2 reduces proliferation, migration and invasion of gastric cancer cells to limit metastasis**. 2015, **10**(2):e0115968.

77. Pfeffer S, Burbaum L, Unverdorben P, Pech M, Chen Y, Zimmermann R, Beckmann R, Förster FJNc: **Structure of the native Sec61 protein-conducting channel**. 2015, **6**:8403.

78. Popow J, Jurkin J, Schleiffer A, Martinez JJN: **Analysis of orthologous groups reveals archease and DDX1 as tRNA splicing factors**. 2014, **511**(7507):104.

79. Zhang Z, Shi Q, Liu Z, Sturgis EM, Spitz MR, Wei QJCE, Biomarkers P: **Polymorphisms of methionine synthase and methionine synthase reductase and risk of squamous cell carcinoma of the head and neck: a case-control analysis**. 2005, **14**(5):1188-1193.

80. Richnau N, Aspenström PJJoBC: **Rich, a rho GTPase-activating protein domain-containing protein involved in signaling by Cdc42 and Rac1**. 2001, **276**(37):35060-35070.

81. Miura K, Hirai M, Kanai Y, Kurosawa YJG: **Organization of the human gene for nucleobindin (NUC) and its chromosomal assignment to 19q13. 2–q13. 4**. 1996, **34**(2):181-186.

82. Demarchi F, Bertoli C, Copetti T, Eskelinen E-L, Schneider CJA: **Calpain as a novel regulator of autophagosome formation**. 2007, **3**(3):235-237.

83. Baertling F, Sanchez‐Caballero L, van den Brand M, Fung CW, Chan SS, Wong VN, Hellebrekers D, de Coo I, Smeitink J, Rodenburg RJCg: **NDUFA9 point mutations cause a variable mitochondrial complex I assembly defect**. 2018, **93**(1):111-118.

84. Genschik P, Billy E, Swianiewicz M, Filipowicz WJTEJ: **The human RNA 3′‐terminal phosphate cyclase is a member of a new family of proteins conserved in Eucarya, Bacteria and Archaea**. 1997, **16**(10):2955-2967.

85. Olive PLJA: **Endogenous DNA breaks: γH2AX and the role of telomeres**. 2009, **1**(2):154.

86. Deng Y-F, Zhou D-N, Pan Z-Y, Yin P: **Aberrant SATB1 expression is associated with Epstein-Barr virus infection, metastasis and survival in human nasopharyngeal cells and endemic nasopharyngeal carcinoma**. *International journal of clinical and experimental pathology* 2014, **7**(5):2454.

87. Chew YC, Adhikary G, Wilson GM, Xu W, Eckert RL: **Sulforaphane induction of p21Cip1 cyclin-dependent kinase inhibitor expression requires p53 and Sp1 transcription factors and is p53-dependent**. *Journal of Biological Chemistry* 2012, **287**(20):16168-16178.

88. Jackson JG, Pereira-Smith OM: **p53 is preferentially recruited to the promoters of growth arrest genes p21 and GADD45 during replicative senescence of normal human fibroblasts**. *Cancer research* 2006, **66**(17):8356-8360.

89. Shankar DB, Cheng JC, Kinjo K, Federman N, Moore TB, Gill A, Rao NP, Landaw EM, Sakamoto KM: **The role of CREB as a proto-oncogene in hematopoiesis and in acute myeloid leukemia**. *Cancer cell* 2005, **7**(4):351-362.
